# Effects of Dialysate Calcium Concentration on Bone, Mineral Metabolism, and Vascular Outcomes in Maintenance Hemodialysis: A Systematic Review and Meta-Analysis

**DOI:** 10.3390/diagnostics16193181

**Published:** 2026-09-30

**Authors:** Ibrahim Alabid, Wasim Iyad Ibrahim Alghoul, Malak Abedi, Mohamed El-Tanani, Mohamedanas Mohamedfaruk Patni, Syed Arman Rabbani, Huthaifa Raid Alabduli, Shams Haidar Abbas Al-Khafaji, Haya Hicham Aljarrah, Khaled Walid Elayyan, Sumaila Firoz, Mohammed Mahmood Ali

**Affiliations:** 1 RAK College of Medical Sciences, RAK Medical and Health Sciences University, Ras Al Khaimah P.O. Box 11172, United Arab Emirates; ibrahim.21901097@rakmhsu.ac.ae (I.A.); wasim.21901111@rakmhsu.ac.ae (W.I.I.A.); malak.abedi0320@gmail.com (M.A.); haya.23901028@rakmhsu.ac.ae (H.H.A.); khaled.22901106@rakmhsu.ac.ae (K.W.E.); sumaila.25901093@rakmhsu.ac.ae (S.F.); mohammed.23901054@rakmhsu.ac.ae (M.M.A.); 2 RAK College of Pharmacy, RAK Medical and Health Sciences University, Ras Al Khaimah P.O. Box 11172, United Arab Emirates; eltanani@rakmhsu.ac.ae; 3 Department of Community Medicine, RAK College of Medical Sciences, RAK Medical and Health Sciences University, Ras Al Khaimah P.O. Box 11172, United Arab Emirates; 4 College of Medicine, University of Sharjah, Sharjah P.O. Box 32223, United Arab Emirates; u22103640@sharjah.ac.ae; 5 College of Medicine, Dubai Medical University, Dubai P.O. Box 20170, United Arab Emirates; sha20240509@dmu.ae

**Keywords:** hemodialysis, dialysate calcium, chronic kidney disease–mineral and bone disorder, bone mineral density, parathyroid hormone, vascular calcification, calcium balance, systematic review and meta-analysis

## Abstract

**Background/Objectives**: The optimal dialysate calcium concentration in maintenance hemodialysis remains uncertain because reducing calcium exposure may limit calcium loading but can also stimulate parathyroid hormone secretion and impair intradialytic cardiovascular stability. We evaluated randomized evidence across bone, mineral metabolism, vascular, and acute safety outcomes. **Methods**: PubMed, the Cochrane Central Register of Controlled Trials (CENTRAL), and Scopus were searched from database inception through 10 July 2026, with targeted citation and publication-status updating through 1 August 2026. Randomized parallel, crossover, factorial, and multi-period trials comparing dialysate calcium concentrations in adults receiving hemodialysis or hemodiafiltration were eligible. Acute and chronic outcomes were analyzed separately. Pooled estimates used inverse-variance random-effects models with DerSimonian–Laird estimation and modified Hartung–Knapp confidence intervals. **Results**: Twenty-four randomized parent trials were included. Lower dialysate calcium reduced end-of-dialysis ionized calcium (paired mean difference, −0.193 mmol/L; 95% CI, −0.258 to −0.128), with substantial heterogeneity. Point estimates for chronic parathyroid hormone and total alkaline phosphatase were higher and serum calcium lower, but all confidence intervals crossed the null. No clear effects were demonstrated for phosphate, calcium–phosphate product, lumbar bone mineral density, coronary artery calcification, or carotid intima–media thickness. Acute parathyroid hormone, blood pressure, calcium-balance, and QTc estimates suggested greater parathyroid stimulation, lower hemodynamic measures, less calcium gain, and longer QTc with lower calcium, but were imprecise. Adverse events were unsuitable for pooling. **Conclusions**: Lower dialysate calcium consistently lowers postdialysis ionized calcium, but its long-term skeletal and vascular effects remain uncertain. No single concentration appears universally optimal; prescription should be individualized according to mineral metabolism phenotype, cardiovascular tolerance, dialysis modality, and concomitant therapy.

## 1. Introduction

Chronic kidney disease–mineral and bone disorder (CKD–MBD) is a systemic complication of kidney failure characterized by abnormalities in mineral metabolism, skeletal remodeling, and extraskeletal calcification. In patients receiving maintenance hemodialysis, renal osteodystrophy may manifest as low-, normal-, or high-turnover bone disease, and distinguishing these phenotypes is clinically important because treatments that suppress excessive turnover may aggravate pre-existing adynamic bone disease. Bone histomorphometry remains the reference standard for defining turnover, mineralization, and bone volume, but its invasive nature restricts routine use. Clinical assessment therefore depends largely on parathyroid hormone (PTH) and biochemical bone turnover markers. However, intact PTH and bone-specific alkaline phosphatase demonstrate only moderate diagnostic discrimination when compared with bone biopsy, and, although combinations of biomarkers may improve classification, they do not fully replace histomorphometric assessment [1,2].

The skeletal consequences of CKD–MBD extend beyond abnormal biochemical measurements. Patients receiving hemodialysis experience substantial fracture risk, but identifying those at greatest risk remains difficult because bone mineral density (BMD), bone turnover, and clinical risk factors capture different dimensions of skeletal fragility. In CKD stage 5D, both low and high PTH concentrations and elevated bone-specific alkaline phosphatase have been associated with incident fractures, whereas the predictive performance of BMD varies according to anatomical site and underlying turnover status [3]. The Fracture Risk Assessment Tool has also demonstrated utility for identifying hemodialysis patients at increased risk of major osteoporotic fractures, although prediction remains imperfect [4]. Furthermore, longitudinal evidence indicates that femoral-neck BMD is more consistently associated with subsequent fractures and mortality than BMD measured at several other skeletal sites, suggesting that site selection and clinical context are important when interpreting densitometric findings in this population [5].

Skeletal disease in hemodialysis is also closely connected to cardiovascular pathology. Vertebral fractures and vascular calcification frequently coexist, supporting the concept of an integrated bone–vascular axis rather than two independent complications of kidney failure [6]. Malnutrition and inflammation may further amplify this relationship, as prospective data have associated these abnormalities with increased risks of both fractures and cardiovascular events [7]. Imaging studies similarly demonstrate an inverse relationship between lumbar volumetric BMD and abdominal aortic calcification, with osteoporosis associated with more severe vascular calcification [8]. Biochemical markers may also reflect this shared risk: in a large nationwide dialysis cohort, higher serum alkaline phosphatase was independently associated with mortality and new hip fractures, although its prognostic relationship varied according to accompanying PTH concentrations [9]. These findings suggest that interventions altering calcium exposure and bone turnover may simultaneously influence skeletal and vascular outcomes.

Dialysate calcium concentration is therefore a clinically important and readily adjustable component of the hemodialysis prescription. Historically, relatively high concentrations were used to suppress secondary hyperparathyroidism; however, increasing concern regarding calcium loading and extraosseous calcification encouraged a shift toward lower-calcium dialysate [10]. This change creates a therapeutic trade-off. A lower concentration may reduce calcium exposure and lessen suppression of skeletal turnover, but it may also promote negative sessional calcium balance and stimulate PTH secretion. Conversely, a higher concentration may improve calcium transfer and suppress PTH while increasing the potential for a positive calcium balance. Importantly, the biological effect of a prescribed concentration is not uniform: direct effluent measurements show that calcium balance varies with the serum-to-dialysate calcium gradient, treatment duration, PTH concentration, concomitant calcium-containing phosphate binders, and other patient-level factors. Even dialysate calcium of 1.25 mmol/L may produce a positive sessional balance in some patients, reinforcing the need to interpret the prescription within the broader clinical and biochemical context [10].

Several receptor- and cell-level mechanisms help explain the skeletal, hormonal, and cardiovascular effects of dialysate calcium. Extracellular ionized calcium is sensed by the calcium-sensing receptor (CaSR) on parathyroid chief cells; lower ionized calcium reduces CaSR activation and stimulates parathyroid hormone (PTH) secretion, whereas higher ionized calcium suppresses PTH release. Acute changes in ionized calcium may also influence vascular tone through calcium entry via L-type voltage-dependent calcium channels in vascular smooth muscle and may affect myocardial contractility. In bone, sustained changes in PTH influence remodeling through osteoblast-lineage and stromal-cell signaling, including regulation of the RANK/RANKL/osteoprotegerin pathway that controls osteoclast differentiation and activity. Persistent PTH excess therefore favors high-turnover bone remodeling, whereas sustained PTH suppression, particularly in the setting of greater calcium exposure, may contribute to low-turnover or adynamic bone disease.

Previous systematic reviews have addressed selected aspects of dialysate calcium prescription. Yoshikawa et al. synthesized seven randomized controlled trials involving 622 patients receiving maintenance hemodialysis for at least six months and found that lower dialysate calcium reduced serum calcium while increasing intact PTH, whereas findings for other mineral-bone and vascular outcomes were less consistent [11]. More recently, Kamei et al. identified 19 randomized controlled trials and quantitatively synthesized eight, with particular emphasis on cardiovascular calcification, mortality, vascular structure and function, and biochemical outcomes [12]. However, these previous syntheses did not provide the same integrated assessment of chronic skeletal and vascular outcomes together with acute ionized-calcium, calcium-balance, hormonal, hemodynamic, and electrophysiological responses. The present review therefore extends previous evidence syntheses by evaluating these competing long-term and acute effects within a unified outcome-specific framework.

Despite its clinical relevance, the optimal dialysate calcium concentration remains uncertain. Randomized studies have evaluated heterogeneous concentrations, dialysis modalities, treatment durations, and outcome definitions. Longer-term trials have examined BMD, bone histomorphometry, PTH, biochemical turnover markers, mineral metabolism, and vascular calcification, whereas acute crossover studies have focused on ionized calcium, calcium balance, blood pressure, arterial function, and cardiac repolarization. Consequently, the evidence has not been integrated adequately across the competing skeletal, vascular, and short-term physiological effects of dialysate calcium selection. Notably, although the present search was updated through 2026, the most recent eligible randomized parent trial was published in 2018, highlighting the lack of contemporary randomized evidence in this field.

Accordingly, this systematic review and outcome-specific meta-analysis of randomized trials was conducted to (1) determine the effects of lower versus higher dialysate calcium concentrations on BMD, bone turnover, and chronic mineral metabolism in adults receiving maintenance hemodialysis; (2) assess their effects on vascular calcification and vascular structure or function; and (3) characterize acute calcium, hormonal, hemodynamic, and electrophysiological responses relevant to treatment safety. By integrating these outcomes within a unified bone–vascular framework, this review aims to clarify the balance of benefits and risks and support a more individualized approach to dialysate calcium prescription.

## 2. Materials and Methods

### 2.1. Study Design, Reporting, and Registration

This systematic review and outcome-specific meta-analysis evaluated randomized trials comparing different dialysate calcium concentrations in adults receiving maintenance hemodialysis and was conducted and reported in accordance with the Preferred Reporting Items for Systematic Reviews and Meta-Analyses (PRISMA) 2020 statement [13].

The review protocol was registered in the International Prospective Register of Systematic Reviews (PROSPERO; registration number CRD420261474874). The protocol defined the eligibility criteria, outcome domains, study-selection procedures, data-extraction methods, risk-of-bias assessment, and statistical analyses before completion of the quantitative synthesis. Any deviations from the registered protocol were documented and justified.

The eligible evidence included parallel-group, crossover, factorial, Latin-square, and randomized multi-period designs. Because physiological effects measured during a single dialysis session are not directly equivalent to those resulting from sustained exposure, acute mechanistic and safety outcomes were analyzed separately from chronic bone, mineral metabolism, and vascular outcomes. Each outcome was synthesized independently rather than combined into a composite estimate.

### 2.2. Eligibility Criteria and Outcome Framework

Studies were eligible if they enrolled adults receiving maintenance hemodialysis or hemodiafiltration and randomly assigned participants, treatment periods, or dialysis sessions to two or more dialysate calcium concentrations. Randomized comparisons conducted using closely related extracorporeal modalities, including predilution hemofiltration, were eligible for modality-inclusive sensitivity analyses but were not included in the principal conventional hemodialysis or hemodiafiltration analyses unless considered clinically comparable.

Eligible designs included parallel-group randomized controlled trials, randomized crossover trials, randomized factorial trials, Latin-square trials, and randomized multi-period comparisons. Studies were excluded if they were observational, retrospective, nonrandomized, conducted exclusively in pediatric or peritoneal-dialysis populations, or did not permit the independent effect of dialysate calcium to be determined.

Trials in which calcium concentration varied simultaneously with another dialysate component or treatment were included only when the effect of dialysate calcium could be isolated or when the cointervention was balanced across the relevant treatment groups. Comparisons in which the calcium effect remained confounded by another intervention were excluded from the principal quantitative synthesis and, when informative, summarized separately.

Multiple reports arising from the same randomized population were linked and treated as a single parent study. Companion and secondary publications were used to supplement the primary report but did not contribute duplicate participants or repeated outcome data to the same synthesis.

Outcomes were organized into three prespecified domains. The bone and chronic mineral metabolism domain included bone mineral density, bone histomorphometry, intact parathyroid hormone, total and bone-specific alkaline phosphatase, other bone turnover markers, serum calcium, serum phosphate, and the calcium–phosphate product. The long-term vascular domain included coronary artery calcification, carotid intima–media thickness, pulse-wave velocity, arterial compliance, and other measures of vascular structure or stiffness. The acute mechanistic and safety domain included ionized calcium, sessional calcium mass balance, acute parathyroid hormone responses, systolic blood pressure, mean arterial pressure, QT or corrected QT interval, intradialytic symptoms, and adverse events.

For chronic outcomes reported at multiple time points, the longest clinically relevant follow-up that remained comparable with the other contributing studies was selected. When several assessments were reported within the same follow-up window, the measurement closest to the prespecified target time point was used. For acute studies, measurements obtained at the end of dialysis or at the prespecified immediate postdialysis assessment were prioritized. Acute and chronic measurements were not combined in the same synthesis.

### 2.3. Information Sources and Search Strategy

PubMed, the Cochrane Central Register of Controlled Trials (CENTRAL) via the Cochrane Library, and Scopus were searched from database inception through 10 July 2026. No restrictions were applied according to publication year or language. The searches retrieved 387 records from PubMed, 156 from CENTRAL, and 676 from Scopus, yielding 1219 database records before deduplication. The search strategies combined controlled-vocabulary and free-text terms related to maintenance hemodialysis, haemodialysis, hemodiafiltration, hemofiltration, dialysate or replacement-fluid calcium concentration, and randomized or comparative intervention designs.

The reference lists of eligible trials and previous systematic reviews were examined manually. Targeted backward and forward citation tracking was undertaken for key trials and reviews, and publisher records and related reports were cross-checked where necessary to identify companion publications, secondary outcome reports, corrections, and potentially eligible studies not retrieved through the electronic searches. A final targeted citation and publication-status check was completed on 1 August 2026. Dedicated clinical trial registries, including ClinicalTrials.gov and the WHO International Clinical Trials Registry Platform, were not systematically searched.

The complete, reproducible search strategy for each database, including search terms, field tags, limits, database-specific syntax, and search dates, is provided in Supplementary Table S1.

### 2.4. Study Selection and Data Collection

After duplicate records were removed, two reviewers independently screened the titles and abstracts of all retrieved records. Reports considered potentially eligible by either reviewer were obtained in full text and independently evaluated against the prespecified eligibility criteria. Disagreements were resolved through discussion and, when consensus could not be reached, by consultation with a third reviewer. Reasons for exclusion at the full-text stage were recorded. No automated or machine-learning system was used to make eligibility decisions. The complete study-selection process, including record identification, deduplication, screening, full-text eligibility assessment, exclusions, and final study inclusion, is summarized in Figure 1.

Data were collected using a standardized and piloted extraction form. Two reviewers independently extracted or verified information from each eligible report. The collected variables included publication year, country, study design, number and location of participating centers, dialysis modality, eligibility criteria, participant characteristics, sample size, dialysate calcium concentrations, treatment duration, treatment sequence, washout period, concomitant therapies, outcome definitions, measurement methods, follow-up time points, attrition, adverse events, study funding, and reported conflicts of interest.

For crossover and randomized multi-period studies, additional information was collected on treatment-sequence randomization, period duration, washout, paired sample size, potential carryover, period effects, and whether the original analysis accounted for within-participant correlation.

Numerical data were obtained from the original full-text reports, tables, figures, appendices, and Supplementary Materials. When several publications described the same parent trial, information was combined across reports and assigned to one study record.

All extracted values and calculated effect estimates were checked against the original source documents before synthesis. Conflicting values between the abstract, main text, tables, figures, or Supplementary Materials were resolved using the most complete outcome-specific report, and the selected values and rationales were documented.

When required numerical data were not reported directly, they were calculated only when sufficient compatible information was available from standard errors, confidence intervals, exact *p*-values, or other summary statistics. No unreported outcome value was otherwise assumed.

### 2.5. Risk-of-Bias and Certainty-of-Evidence Assessments

Two reviewers independently assessed risk of bias for each relevant result using the Cochrane Risk of Bias 2 (RoB 2) tool. The crossover-trial version of RoB 2 was used where applicable. The assessment considered bias arising from the randomization process, deviations from intended interventions, missing outcome data, measurement of the outcome, and selection of the reported result. Each result was classified as having low risk of bias, some concerns, or high risk of bias. Disagreements were resolved through discussion and, when necessary, third-reviewer adjudication.

Complete result-specific RoB 2 judgments for outcomes contributing to the principal quantitative syntheses are provided in Supplementary Table S3. Figure 2 is retained as a condensed study-level overview of recurring risk-of-bias patterns across the included trials and does not replace the result-specific assessments. The outcome-specific RoB 2 judgments were used to inform the risk-of-bias domain of the corresponding GRADE assessments. An overall RoB 2 judgment of “some concerns” for an individual result did not automatically result in downgrading the certainty of evidence; GRADE risk-of-bias judgments were made at the outcome/body-of-evidence level, considering the nature and likely impact of the identified limitations across contributing results.

For crossover trials, particular attention was given to randomization of treatment sequence, adequacy of washout, potential period and carryover effects, missing paired observations, and whether the original statistical analysis preserved the paired design. Long-duration crossover studies evaluating structural outcomes, such as bone mineral density, were considered particularly vulnerable to residual treatment effects and were not included in the principal parallel-group BMD synthesis when first-period results could not be isolated.

Certainty of evidence for each principal outcome was evaluated using the Grading of Recommendations Assessment, Development and Evaluation (GRADE) approach. Randomized evidence initially received a high-certainty rating and was downgraded when appropriate for risk of bias, inconsistency, indirectness, imprecision, or suspected publication bias. Certainty was classified as high, moderate, low, or very low. Judgments were made at the outcome level rather than by assigning a single overall rating to the review. 

### 2.6. Effect Measures and Data Preparation

Continuous outcomes reported using a common unit were summarized as mean differences with 95% confidence intervals. Paired mean differences were used for crossover comparisons when outcomes were measured in the same unit. When conceptually equivalent outcomes were measured using different scales or imaging approaches, standardized mean differences corrected using Hedges’ g were calculated.

Patient-level binary adverse events were summarized using risk ratios with 95% confidence intervals when event definitions and reporting permitted meaningful quantitative comparison. Repeated session-level events were not analyzed as independent patient-level events because several dialysis sessions could originate from the same participant. Such outcomes were summarized narratively unless the original analysis adequately accounted for clustering.

All effects were coded as the value in the lower dialysate calcium condition minus the corresponding value in the higher dialysate calcium condition. The clinical interpretation of positive and negative estimates was, therefore, defined separately for each outcome.

For parallel-group trials, effects were calculated from final values or changes from baseline according to the form most consistently and completely reported for the relevant outcome. Final-value and change-score effects were combined only when they represented clinically equivalent treatment contrasts on the same measurement scale.

When a change-score standard deviation was unavailable but baseline and final standard deviations were reported, it was reconstructed assuming a within-group pre–post correlation of 0.50. Correlations of 0.25 and 0.75 were examined in sensitivity analyses.

For crossover trials, paired mean differences and paired standard errors were used when reported. When the variance of the within-participant difference was unavailable, it was reconstructed from the treatment-period standard deviations using an assumed within-participant correlation of 0.50. Correlations of 0.25 and 0.75 were examined in sensitivity analyses. Crossover observations were not treated as independent parallel-group observations.

Standard deviations were derived, when necessary, from standard errors, confidence intervals, exact *p*-values, or other compatible summary statistics. Measurement units were harmonized before pooling, including conversion of calcium, phosphate, ionized calcium, calcium mass balance, and calcium–phosphate product measurements to common units.

Total or corrected calcium was not pooled with ionized calcium. Total alkaline phosphatase was not pooled with bone-specific alkaline phosphatase. Arithmetic mean differences in PTH were not combined directly with ratios of geometric means.

For multi-arm and factorial trials, one independent lower-versus-higher calcium contrast was selected for each analysis. Comparisons were selected to isolate the calcium effect, maintain clinical comparability, and avoid repeated use of the same participants. When calcium was varied within factorial combinations of potassium, magnesium, bicarbonate, or another dialysate component, calcium contrasts were matched within the same level of the additional factor whenever possible.

Results that were markedly skewed, reported only as medians without convertible dispersion measures, or otherwise incompatible with the prespecified effect measures were summarized narratively. No participant group was entered more than once in the same pooled analysis.

### 2.7. Data Synthesis and Statistical Analysis

Meta-analysis was undertaken when at least two studies were sufficiently comparable in population, dialysis modality, dialysate calcium contrast, duration of exposure, outcome definition, and measurement method. Study characteristics were reviewed before each synthesis to determine eligibility for pooling. Clinically or methodologically incompatible results were presented individually or synthesized narratively.

Effect estimates were pooled using inverse-variance random-effects models because clinical and methodological heterogeneity among the included trials was anticipated. Between-study variance was estimated using the DerSimonian–Laird method, an approach also applied in contemporary clinical systematic reviews and meta-analyses [14].

Because most outcome-specific syntheses contained a small number of studies, 95% confidence intervals and tests of pooled effects were calculated using a modified Hartung–Knapp approach. A safeguard was applied to prevent the modified variance estimate from becoming smaller than the corresponding conventional random-effects variance estimate.

**Figure 2:**
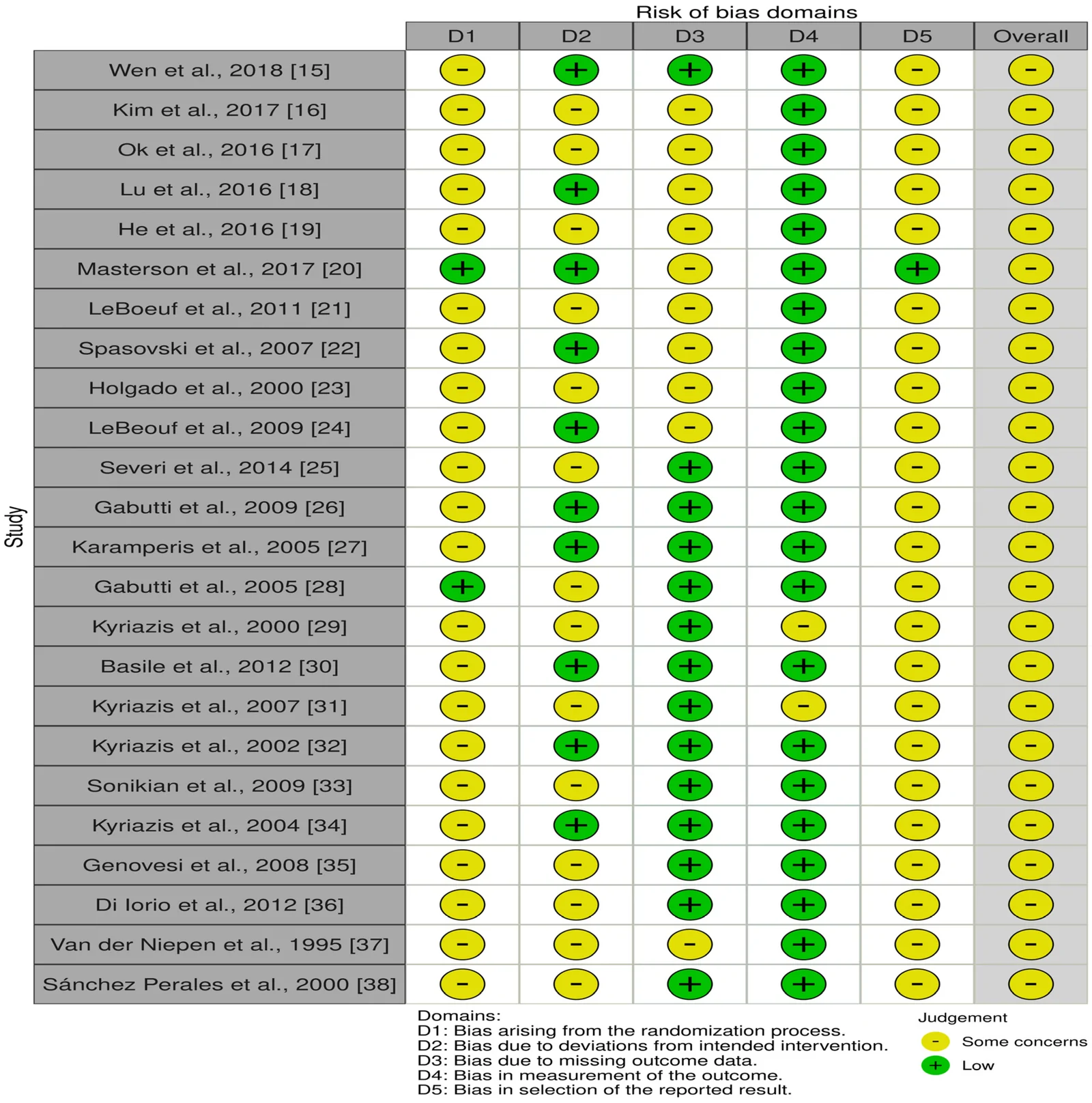
Condensed study-level overview of risk-of-bias patterns across the 24 included randomized trials using the Cochrane Risk of Bias 2 (RoB 2) framework. D1, bias arising from the randomization process; D2, bias due to deviations from intended interventions; D3, bias due to missing outcome data; D4, bias in measurement of the outcome; and D5, bias in selection of the reported result. Green symbols indicate low risk of bias, whereas yellow symbols indicate some concerns. The figure is provided as a concise overview of recurring domain-level concerns across trials; complete result-specific RoB 2 judgments for outcomes contributing to the principal quantitative syntheses are provided in Supplementary Table S3. For crossover trials, treatment sequence, period, and carryover considerations were incorporated into the harmonized graphical overview [15,16,17,18,19,20,21,22,23,24,25,26,27,28,29,30,31,32,33,34,35,36,37,38].

Statistical heterogeneity was evaluated using Cochran’s Q statistic, the between-study variance estimate τ^2^, and the I^2^ statistic. These measures were interpreted together with the magnitude, direction, and clinical consistency of the individual study effects rather than according to rigid numerical thresholds alone.

A 95% prediction interval was calculated for syntheses containing at least three clinically comparable studies, when estimable. Prediction intervals were not calculated for two-study meta-analyses.

Potential sources of heterogeneity were considered according to study design, dialysis modality, duration of calcium exposure, magnitude of the dialysate calcium contrast, participant phenotype, baseline PTH or bone turnover status, and the presence of relevant cointerventions. Formal meta-regression was not performed because too few studies contributed to the individual outcome-specific syntheses for a reliable multivariable analysis.

Individual study estimates, 95% confidence intervals, random-effects weights, and pooled effects were displayed using forest plots. Two-sided *p*-values below 0.05 were considered statistically significant; however, interpretation emphasized the magnitude and precision of the effect, clinical relevance, consistency across trials, and certainty of evidence rather than statistical significance alone.

### 2.8. Sensitivity Analyses, Reporting Bias, and Data Availability

Sensitivity analyses were selected according to the design and clinical characteristics of each outcome. These included restriction to conventional hemodialysis or hemodiafiltration, exclusion of atypical extracorporeal modalities, exclusion of studies with important cointerventions, removal of studies judged to be at high risk of bias, alternative within-participant correlations for crossover studies, alternative pre–post correlations for reconstructed change-score variances, and leave-one-out analyses for syntheses containing at least three studies.

To assess the robustness of the findings to the choice of between-study heterogeneity estimator, all primary quantitative syntheses were additionally repeated using restricted maximum likelihood (REML) estimation of τ^2^. Study inclusion, effect measures, effect direction, variance reconstruction, crossover assumptions, and the modified Hartung–Knapp standard-error safeguard were otherwise retained exactly as in the primary DerSimonian–Laird analyses. Results of the REML sensitivity analyses are presented in Supplementary Table S2.

The principal acute ionized-calcium analysis was restricted to conventional hemodialysis or hemodiafiltration. A separate modality-inclusive sensitivity analysis incorporated other eligible extracorporeal modalities, including predilution hemofiltration.

The principal BMD synthesis was restricted to parallel-group evidence. Long-duration crossover QCT evidence was designated as nonprimary because persistent skeletal effects and treatment carryover could not be excluded. These studies were presented separately or included only in sensitivity analyses and were not used as the principal basis for conclusions regarding BMD.

The potential influence of missing results and selective nonreporting was evaluated by comparing the outcomes and analyses described in the methods of each trial with those reported in the results, examining companion publications, and determining whether findings were reported only for selected time points, analyses, or treatment comparisons. Funnel plots and formal tests of small-study effects were not performed because no homogeneous, outcome-specific synthesis included at least ten independent studies. The possibility of publication bias was considered qualitatively and incorporated into the outcome-specific GRADE assessments.

The complete database search strategies, sensitivity analyses, and outcome-specific risk-of-bias assessments are provided in the Supplementary Materials.

## 3. Results

### 3.1. Study Selection

The electronic database searches identified 1219 records, comprising 387 records from PubMed/MEDLINE, 156 from CENTRAL, and 676 from Scopus. After the removal of 438 duplicate records, 781 records underwent title and abstract screening. Of these, 700 records were excluded, and 81 reports were sought for full-text retrieval. Two reports could not be retrieved, leaving 79 reports for full-text eligibility assessment. Fifty-five reports were excluded: 21 because of a nonrandomized or observational study design, 12 because the independent effect of dialysate calcium could not be isolated or was confounded by another intervention, 7 because of an ineligible population or dialysis modality, 9 because they did not provide relevant or extractable randomized outcome data, and 6 because information was insufficient for eligibility or quantitative assessment. Twenty-four randomized parent trials were ultimately included in the systematic review [15,16,17,18,19,20,21,22,23,24,25,26,27,28,29,30,31,32,33,34,35,36,37,38].

### 3.2. Characteristics of Included Studies

The 24 parent trials were published between 1995 and 2018 and collectively enrolled 1336 participants. Ten studies used parallel-group randomized designs, whereas the remaining trials used crossover, factorial, Latin-square, or other randomized multi-period designs. The duration of exposure ranged from a single dialysis session to 24 months.

Most studies evaluated conventional maintenance hemodialysis. One trial investigated hemodiafiltration, one evaluated predilution hemofiltration, and one examined alternate-night nocturnal hemodialysis. The evaluated dialysate or infusate calcium concentrations ranged from 1.00 to 2.00 mmol/L, although the most frequently investigated comparison involved a lower concentration of approximately 1.25 mmol/L and a higher concentration of 1.50 or 1.75 mmol/L.

The longer-term trials evaluated BMD, bone histomorphometry, biochemical markers of bone turnover, mineral metabolism, coronary artery calcification, carotid intima–media thickness, and arterial stiffness. The acute crossover trials examined ionized calcium, calcium mass balance, PTH responses, blood pressure, arterial function, QT and QTc intervals, intradialytic symptoms, and other safety outcomes. Detailed characteristics of the 24 included randomized parent trials are summarized in Table 1.

Several design-specific issues influenced the eligibility of individual results for quantitative synthesis. These included concurrent variation in magnesium, potassium, or bicarbonate concentrations in factorial trials; treatment-period cointerventions; absence of washout in long-duration crossover studies; and incomplete reporting of paired variances. Accordingly, primary, sensitivity, and narrative evidence were distinguished before pooling.

### 3.3. Risk of Bias and Certainty of Evidence

Figure 2 provides a condensed study-level overview of recurring risk-of-bias patterns across the 24 included randomized trials. The formal RoB 2 assessments used for evidence synthesis were performed at the result level, and complete domain-level judgments for results contributing to the principal quantitative syntheses are presented in Supplementary Table S3. Across these result-specific assessments, most individual domains were judged to be at low risk of bias, while recurrent concerns related to incomplete reporting of sequence generation or allocation procedures, deviations from intended interventions, missing outcome data, and selection of the reported result. All results contributing to the principal quantitative syntheses were judged as having some concerns overall; none was judged to be at high risk of bias. For crossover results, treatment-sequence randomization, washout, potential period or carryover effects, missing paired observations, and preservation of the paired design in the original analysis were additionally considered. These result-specific assessments informed the risk-of-bias judgments used in the outcome-level GRADE evaluations presented in Table 2.

The certainty of evidence varied across outcomes (Table 2). End-of-dialysis ionized calcium was judged to provide moderate-certainty evidence, reflecting a consistent direction of effect and confidence and prediction intervals that excluded no difference, despite substantial heterogeneity in effect magnitude. Evidence for carotid intima–media thickness and postdialysis systolic blood pressure was rated as low certainty. Evidence for lumbar BMD, chronic PTH, total ALP, serum calcium, phosphate, calcium–phosphate product, coronary artery calcification, acute PTH response, calcium mass balance, mean arterial pressure, and QTc was rated as very low certainty because of combinations of risk of bias, inconsistency, indirectness, and imprecision. No outcome was downgraded specifically for publication bias, although publication bias could not be excluded because all outcome-specific syntheses contained fewer than 10 independent studies.

### 3.4. Bone Outcomes

#### 3.4.1. Bone Mineral Density

Two parallel-group trials involving 62 participants contributed to the exploratory lumbar BMD meta-analysis [20,38]. The pooled estimate showed no clear difference between lower and higher dialysate calcium concentrations, with a Hedges’ g of 0.04 (95% CI, −3.20 to 3.28; *p* = 0.905). No statistical heterogeneity was detected (I^2^ = 0%; τ^2^ = 0). However, the confidence interval was very wide, reflecting the small number of participants and methodological differences between the contributing trials, including the use of different BMD measurement approaches and alternate-night nocturnal hemodialysis in one study. The exploratory lumbar BMD meta-analysis is presented in Figure 3A.

QCT percentage-change results were not pooled in the primary analysis. In the parallel trial by Sánchez Perales et al. [38], lumbar QCT BMD decreased by 15.0% in the lower-calcium group and increased by 1.28% in the higher-calcium group, producing a mean difference of −16.28 percentage points (95% CI, −29.78 to −2.78).

In the long-duration crossover study by Van der Niepen et al. [37], QCT BMD increased by 2.1% during exposure to lower dialysate calcium and by 7.0% during exposure to higher calcium. The paired mean difference was −4.90 percentage points (95% CI, −10.57 to 0.77). This estimate was retained only as nonprimary evidence because each treatment period lasted six months, no washout period was used, and the skeletal effect of the first treatment could have persisted into the second period. The combined crossover QCT estimate was not used in the abstract or principal conclusions.

#### 3.4.2. Bone Histomorphometry

Repeat bone-biopsy data were available from one 24-month randomized trial, in which paired biopsies were obtained from 108 participants [17]. Because no second compatible randomized study was identified, the histomorphometric results were synthesized narratively.

Bone formation rate increased from 1.03 to 3.24 mm^3^/cm^2^/year in the lower-calcium group and from 1.41 to 1.93 mm^3^/cm^2^/year in the higher-calcium group. Activation frequency increased from 0.22 to 0.67 per year with lower calcium and from 0.29 to 0.41 per year with higher calcium. Bone volume increased from 18.6% to 21.3% in the lower-calcium group but decreased from 18.9% to 18.0% in the higher-calcium group.

Cortical porosity increased in both treatment groups, from 2.47% to 14.78% with lower dialysate calcium and from 2.69% to 10.88% with higher dialysate calcium. These findings suggested substantial changes in skeletal remodeling during follow-up, but a pooled treatment effect could not be estimated from a single trial.

#### 3.4.3. Chronic Parathyroid Hormone

Four trials contributed arithmetic PTH values to the primary chronic synthesis [18,22,23,38]. Lower dialysate calcium was associated with a numerically higher chronic PTH concentration, with a pooled mean difference of 39.61 pg/mL. The modified Hartung–Knapp confidence interval included no difference (95% CI, −5.38 to 84.59; *p* = 0.068).

Heterogeneity was substantial (I^2^ = 67.3%; τ^2^ = 350.85), and the 95% prediction interval ranged from −57.66 to 136.87 pg/mL. The variation between trials was consistent with differences in baseline PTH concentrations, bone turnover phenotypes, treatment duration, and concomitant CKD–mineral and bone disorder therapies. PTH results reported as geometric means, medians, time-averaged values, or isolated change scores were not combined with the arithmetic final-value synthesis. The pooled chronic PTH analysis is presented in Figure 3B.

#### 3.4.4. Alkaline Phosphatase and Other Bone Turnover Markers

Five trials contributed total ALP data [16,20,22,23,38]. The pooled mean difference was 21.20 U/L in the direction of a higher total ALP concentration with lower dialysate calcium, although the confidence interval crossed the null (95% CI, −6.04 to 48.44; *p* = 0.097). Moderate heterogeneity was observed (I^2^ = 49.9%; τ^2^ = 171.79), and the prediction interval ranged from −29.15 to 71.55 U/L.

Total ALP was not combined with bone-specific ALP because the two measurements do not represent interchangeable outcomes. In one six-month trial, bone-specific ALP was 35.6 U/L in the lower-calcium group and 22.5 U/L in the higher-calcium group [22]. Another trial reported CTX concentrations of 1696 and 1482 ng/L and P1NP concentrations of 382 and 332 µg/L in the lower- and higher-calcium groups, respectively [21]. These marker-specific results were summarized narratively because no second sufficiently compatible randomized study reported the same outcome using a comparable assay and time point. The pooled total ALP analysis is presented in Figure 3C.

### 3.5. Chronic Mineral Metabolism Outcomes

Four trials contributed total or corrected serum calcium data [16,18,19,22]. Lower dialysate calcium was associated with a pooled mean difference of −0.103 mmol/L, indicating a lower chronic serum calcium concentration relative to higher dialysate calcium. The modified Hartung–Knapp confidence interval included no difference (95% CI, −0.262 to 0.055; *p* = 0.130). Heterogeneity was considerable (I^2^ = 79.7%; τ^2^ = 0.0076), and the prediction interval ranged from −0.532 to 0.325 mmol/L.

Five trials contributed serum phosphate data [16,18,19,22,23]. The pooled mean difference was −0.085 mmol/L (95% CI, −0.465 to 0.295; *p* = 0.570), providing no clear evidence of a chronic difference between lower and higher dialysate calcium prescriptions. Heterogeneity was high (I^2^ = 87.5%; τ^2^ = 0.0796), and the prediction interval extended from −1.082 to 0.913 mmol/L.

Three trials reported directly calculated calcium–phosphate products in compatible units [16,18,22]. The pooled mean difference was 0.117 mmol^2^/L^2^ (95% CI, −0.876 to 1.110; *p* = 0.662). Heterogeneity was substantial (I^2^ = 67.0%; τ^2^ = 0.1053), and the prediction interval ranged from −4.942 to 5.176 mmol^2^/L^2^. The pooled chronic serum calcium, phosphate, and calcium–phosphate product analyses are presented in Figure 4A, Figure 4B, and Figure 4C, respectively.

### 3.6. Vascular Outcomes

#### 3.6.1. Coronary Artery Calcification

Two parallel-group trials contributed compatible continuous data for change in coronary artery calcium score [15,17]. The pooled mean difference was −197.06 Agatston units, in the direction of less calcification progression with lower dialysate calcium. However, the modified Hartung–Knapp confidence interval was wide and crossed the null (95% CI, −915.15 to 521.02; *p* = 0.178). Heterogeneity was low to moderate (I^2^ = 25.4%; τ^2^ = 1627.75).

A third randomized trial reported that coronary calcification progressed in both groups and classified participants as progressors according to tertiles of CAC change [16]. That trial found a higher proportion of progressors among participants assigned to lower dialysate calcium and reported an adjusted odds ratio of 5.72 (95% CI, 1.22–26.84). Because the outcome definition and analytical scale differed from the continuous change-score model, this result was not combined directly with the other trials.

The vascular calcification evidence was therefore directionally inconsistent across individual trials. The pooled continuous estimate was imprecise and did not establish that lower dialysate calcium either reduced or accelerated CAC progression. The pooled CAC analysis is presented in Figure 5A.

#### 3.6.2. Carotid Intima–Media Thickness

Two parallel-group trials contributed final cIMT measurements [18,19]. The pooled mean difference was −0.072 mm in favor of lower dialysate calcium, but the modified Hartung–Knapp confidence interval included no difference (95% CI, −0.308 to 0.165; *p* = 0.162). No statistical heterogeneity was detected (I^2^ = 0%; τ^2^ = 0), although this finding should be interpreted cautiously because only two studies contributed. The pooled cIMT analysis is presented in Figure 5B.

#### 3.6.3. Arterial Stiffness

Arterial-stiffness outcomes could not be pooled because the trials used different vascular segments, measurement techniques, time points, and statistical effect measures [19,20,21,24,29,31]. The reported outcomes included carotid–femoral and carotid–radial pulse-wave velocity, augmentation index, central pulse-wave parameters, and arterial compliance.

The acute crossover studies generally showed that higher dialysate calcium increased postdialysis blood pressure and arterial stiffness, although the size of the response differed among studies. The longer-term trials provided less consistent findings, and several reported repeated-measures or mixed-model estimates that could not be converted reliably to a common effect measure. These results were therefore synthesized narratively.

### 3.7. Acute Physiological and Safety Outcomes

#### 3.7.1. Acute Parathyroid Hormone Response

Three randomized crossover trials contributed to the acute PTH synthesis [24,30,34]. Lower dialysate calcium produced a larger acute increase, or less suppression, of PTH, with a pooled paired mean difference of 267.03 pg/mL. The confidence interval was wide and crossed the null (95% CI, −49.16 to 583.22; *p* = 0.068).

Substantial heterogeneity was present (I^2^ = 69.2%; τ^2^ = 10,724.64), and the prediction interval ranged from −1337.34 to 1871.41 pg/mL. The direction of the point estimates was compatible with greater acute parathyroid stimulation under lower-calcium dialysis, but the magnitude of the response varied markedly among the contributing trials. The pooled acute PTH analysis is presented in Figure 6A.

#### 3.7.2. Acute Ionized Calcium

Six conventional hemodialysis or hemodiafiltration crossover trials contributed to the primary end-of-dialysis ionized-calcium analysis [25,28,29,30,31,34]. Lower dialysate calcium produced a significantly lower postdialysis ionized calcium concentration, with a pooled paired mean difference of −0.193 mmol/L (95% CI, −0.258 to −0.128; *p* < 0.001).

Statistical heterogeneity was high (I^2^ = 90.7%; τ^2^ = 0.0027), reflecting differences in calcium gradients, treatment modalities, and study protocols. Nevertheless, all contributing estimates favored a lower postdialysis ionized calcium concentration with the lower-calcium prescription. The 95% prediction interval ranged from −0.351 to −0.034 mmol/L and therefore also excluded no difference.

The modality-inclusive sensitivity analysis added the predilution-hemofiltration trial and included seven studies [25,27,28,29,30,31,34]. The resulting estimate was similar to the primary analysis, with a paired mean difference of −0.202 mmol/L (95% CI, −0.258 to −0.146; *p* < 0.001; I^2^ = 92.4%). The primary conventional hemodialysis/hemodiafiltration ionized-calcium analysis is presented in Figure 6B, while the all-modality sensitivity analysis is shown in Supplementary Figure S1.

#### 3.7.3. Calcium Mass Balance

Two randomized crossover trials reported acute calcium mass balance in units that could be harmonized [25,30]. Lower dialysate calcium was associated with less calcium gain or greater calcium loss, with a pooled, paired mean difference of −11.23 mmol/session. The confidence interval was wide and included no difference (95% CI, −88.40 to 65.94; *p* = 0.316).

Heterogeneity was considerable (I^2^ = 93.7%; τ^2^ = 69.31). The two studies differed in the calcium concentrations examined, dialysis modality, and method of calculating calcium balance. In the three-period crossover trial by Basile et al. [30], calcium mass balance became progressively more positive as dialysate calcium increased, while PTH rose during the lowest-calcium session and declined during the intermediate- and higher-calcium sessions. The pooled acute calcium-balance analysis is presented in Figure 6C.

#### 3.7.4. Hemodynamic Outcomes

Three crossover trials contributed postdialysis systolic blood pressure data [27,28,29]. Lower dialysate calcium was associated with a pooled paired mean difference in postdialysis systolic blood pressure of −8.97 mmHg (95% CI, −20.48 to 2.55; *p* = 0.079). No statistical heterogeneity was detected (I^2^ = 0%; τ^2^ = 0), and the prediction interval ranged from −42.97 to 25.04 mmHg.

Two trials contributed postdialysis mean arterial pressure data [27,29]. The pooled paired mean difference was −8.65 mmHg (95% CI, −33.45 to 16.14; *p* = 0.141), with no detected statistical heterogeneity (I^2^ = 0%; τ^2^ = 0). These findings indicated lower hemodynamic point estimates during lower-calcium dialysis, but the small number of studies resulted in considerable uncertainty around the pooled effects. The pooled postdialysis systolic blood pressure and mean arterial pressure analyses are presented in Figure 6D and Figure 6E, respectively.

#### 3.7.5. QTc Interval

Two randomized crossover trials contributed end-of-dialysis QTc measurements [25,35]. One fixed-potassium stratum from the factorial trial by Genovesi et al. [35] was selected to prevent the same participants from contributing correlated comparisons more than once.

Lower dialysate calcium was associated with a numerically longer end-of-dialysis QTc interval, with a pooled paired mean difference of 27.31 ms. The confidence interval was wide and included no difference (95% CI, −41.82 to 96.43; *p* = 0.125). Heterogeneity was low (I^2^ = 20.5%; τ^2^ = 12.38).

The alternative fixed-potassium stratum produced a result in the same direction and was retained as a sensitivity comparison. Di Iorio et al. [36] could not be included in the pooled QTc analysis because the matched calcium contrasts lacked usable variance estimates, whereas the extractable abstract comparison simultaneously changed calcium, potassium, and bicarbonate concentrations. The pooled end-of-dialysis QTc analysis is presented in Figure 6F.

### 3.8. Adverse Events

Adverse-event definitions, denominators, and reporting methods varied substantially among the included trials, preventing a meaningful pooled safety analysis.

In the 24-month trial by Ok et al. [17], any adverse event occurred in 95 of 212 participants assigned to lower dialysate calcium and 117 of 213 assigned to higher calcium. Serious adverse events occurred in 79 and 97 participants, respectively, while deaths occurred in 31 and 37 participants. Permanent treatment discontinuation occurred in no participant assigned to lower calcium and in 15 participants assigned to higher calcium. Because these outcomes were derived predominantly from a single large trial, they were reported as individual study findings rather than pooled estimates.

In the nocturnal-hemodialysis trial [20], hypocalcemia occurred in 9 of 22 participants assigned to lower dialysate calcium and in 1 of 20 assigned to higher calcium, corresponding to a risk ratio of 8.18 (95% CI, 1.13–58.98). Hypercalcemia occurred in one participant in each group.

Wen et al. [15] reported intradialytic hypotension, hypertension, muscle cramps, and arrhythmias, using dialysis sessions rather than patients as the denominator. These repeated observations were not treated as independent binary events because multiple sessions originated from the same participants. The original trial reported no significant overall difference in the frequency of intradialytic adverse reactions between the calcium groups.

Other crossover studies reported symptoms or hypotensive episodes by treatment session, but paired binary data and within-participant event distributions were generally unavailable. These results were, therefore, summarized narratively.

### 3.9. Sensitivity Analyses

Leave-one-out analyses did not materially change the direction of the primary chronic PTH, total ALP, serum calcium, or phosphate estimates. However, confidence intervals remained wide and generally included no difference after the removal of individual studies.

The acute ionized-calcium result was the most robust pooled finding. Sequential omission of each contributing trial produced pooled mean differences ranging from approximately −0.207 to −0.178 mmol/L, and every modified Hartung–Knapp confidence interval remained below zero. The consistency of this finding indicated that the pooled reduction in postdialysis ionized calcium was not driven by a single study.

Changing the assumed within-participant correlation from 0.50 to 0.25 or 0.75 affected the precision of crossover estimates, but did not materially alter their direction. The long-duration crossover QCT estimate remained unsuitable for inclusion in the primary BMD synthesis under all correlation assumptions because the principal concern was potential carryover rather than the assumed variance alone.

Prediction intervals crossed the null for chronic PTH, total ALP, serum calcium, phosphate, calcium–phosphate product, acute PTH, and systolic blood pressure. Acute ionized calcium was the only pooled analysis for which the prediction interval excluded no difference.

Sensitivity analyses using REML estimation of between-study variance did not materially alter the direction or overall interpretation of any of the 14 primary quantitative syntheses compared with the DerSimonian–Laird models (Supplementary Table S2). Differences in the estimated between-study variance and confidence-interval width were modest for most outcomes and did not change the principal conclusions. For end-dialysis ionized calcium, the pooled effect remained −0.193 mmol/L (95% CI, −0.258 to −0.128), with REML τ^2^ = 0.0029, and the prediction interval remained entirely below the null (−0.345 to −0.041 mmol/L). For outcomes with τ^2^ = 0, including lumbar BMD, cIMT, postdialysis SBP, and postdialysis MAP, use of REML did not alter the between-study variance estimate or the interpretation under the prespecified modified Hartung–Knapp safeguard. Overall, the substantive conclusions were robust to the choice of between-study variance estimator.

### 3.10. Reporting Bias

No homogeneous, outcome-specific synthesis included at least 10 independent studies. Funnel plots and formal tests of small-study effects were therefore not performed. The existing funnel coordinates were retained only for analytical documentation and were not interpreted as evidence for or against publication bias.

The possibility of missing results and selective nonreporting was evaluated qualitatively by comparing the outcomes and analyses described in the methods of each trial with those reported in the results, by examining companion publications, and by identifying outcomes reported only at selected time points or for selected treatment contrasts. Publication bias was also considered within the outcome-specific GRADE assessments. No outcome was downgraded specifically for publication bias because there was no affirmative evidence of small-study effects; however, publication bias could not be excluded because all homogeneous outcome-specific syntheses contained fewer than 10 independent studies.

## 4. Discussion

### 4.1. Principal Findings

This systematic review and outcome-specific meta-analysis synthesized 24 randomized parent trials evaluating the effects of different dialysate calcium concentrations on bone, mineral metabolism, vascular, hemodynamic, and electrophysiological outcomes in adults receiving maintenance hemodialysis. The findings demonstrate that the consequences of altering dialysate calcium depend on the duration of exposure, the outcome being assessed, and the patient’s underlying biochemical and clinical phenotype.

The most consistent quantitative finding was that lower dialysate calcium reduced end-of-dialysis ionized calcium. This association remained statistically significant in the primary analysis restricted to conventional hemodialysis or hemodiafiltration, in the modality-inclusive sensitivity analysis, and after sequential exclusion of individual studies. Lower dialysate calcium also produced numerically greater acute PTH stimulation and lower postdialysis blood pressure estimates. A numerically longer QTc interval was observed with lower calcium, although the estimates for PTH, blood pressure, and QTc remained imprecise.

These acute findings illustrate an important physiological trade-off. Reducing the dialysate-to-blood calcium gradient may limit calcium transfer during dialysis, but it can also increase parathyroid stimulation, reduce vascular tone, and potentially affect ventricular repolarization and intradialytic cardiovascular stability.

The chronic evidence was less definitive. Lower dialysate calcium was associated with numerically higher PTH and total alkaline phosphatase, slightly lower total or corrected serum calcium, and no clear differences in phosphate or the calcium–phosphate product. The exploratory parallel-trial BMD analysis did not demonstrate a clear effect, but its wide confidence interval was insufficient for establishing equivalence. Histomorphometric evidence suggested that reducing dialysate calcium may stimulate bone turnover in patients with low-turnover bone disease, although this finding was derived predominantly from a single trial. Similarly, the pooled coronary artery calcification and carotid intima–media thickness estimates were imprecise, and the individual calcification trials yielded directionally inconsistent results.

Collectively, the evidence does not support one dialysate calcium concentration as uniformly optimal for all patients or outcomes. Dialysate calcium should instead be regarded as a modifiable component of the broader CKD–mineral and bone disorder treatment strategy. Its potential benefits and harms are likely to depend on bone turnover status, serum calcium, PTH trajectory, vascular calcification, cardiovascular stability, dialysis modality and duration, and concomitant therapies.

### 4.2. Bone Mineral Density and Fracture Implications

The absence of a clear pooled BMD effect should not be interpreted as evidence that dialysate calcium has no skeletal consequences. Only two small parallel trials contributed to the exploratory lumbar BMD synthesis, and they differed in dialysis modality and measurement technique. The long-duration crossover evidence could not resolve this uncertainty because skeletal changes induced during the first treatment period may have persisted into the second. The randomized evidence is therefore insufficient to determine whether sustained exposure to lower dialysate calcium prevents or accelerates clinically meaningful bone loss.

This distinction is important because BMD and fracture risk are related but not interchangeable in hemodialysis. Lower metacarpal BMD has been associated with clinical and vertebral fractures, while prospective observations have documented deterioration in both BMD and trabecular bone score during maintenance hemodialysis [39,40]. Vertebral fractures may remain clinically unrecognized, and fracture risk is also influenced by age, dialysis duration, diabetes, nutritional status, falls, PTH abnormalities, and previous fractures [41,42]. These competing determinants make it difficult for small dialysate-calcium trials to demonstrate an independent fracture-prevention effect.

Skeletal risk may also vary by the underlying cause of kidney failure and by the anatomical site or bone compartment assessed. Contemporary cohort data suggest that fracture incidence differs across kidney-disease categories and that cortical, trabecular, axial, and appendicular measurements may not carry identical prognostic information [43]. Trabecular bone score and quantitative ultrasound have also been associated with muscle strength and fracture-related phenotypes, indicating that bone quality and neuromuscular function may provide information beyond conventional areal BMD [44].

The interaction between bone and muscle deserves particular consideration. Sarcopenia, altered bone turnover markers, and low BMD commonly coexist in hemodialysis patients [45]. Greater trunk-muscle density has been associated with higher lumbar volumetric BMD and a lower probability of osteopenia, whereas reduced grip strength and low albumin have been identified as risk factors for CKD-associated osteoporosis [46,47]. Future trials should therefore assess nutritional status, muscle strength, physical performance, falls, and frailty alongside skeletal imaging.

Alternative imaging methods may improve identification of high-risk patients. Vertebral attenuation measured opportunistically on abdominal computed tomography has been independently associated with BMD and may facilitate osteoporosis screening in hemodialysis populations [48]. Combining DXA, quantitative CT, vertebral-fracture assessment, trabecular measures, and biochemical markers may be more informative than relying on a single BMD site.

The reported association between calcium-based phosphate-binder use and a lower prevalence of osteoporosis further illustrates the difficulty of isolating dialysate calcium from total calcium exposure [49]. Dietary intake, calcium-containing binders, vitamin D therapy, calcimimetics, residual kidney function, and dialysate calcium collectively influence skeletal mineral balance. Observational treatment associations are also vulnerable to confounding by indication and cannot establish that increasing calcium exposure improves bone outcomes. Similarly, emerging fracture-prediction models that combine BMD with biochemical markers may improve risk classification but do not determine the optimal dialysate calcium concentration [50].

### 4.3. Bone Turnover and Biochemical Markers

The numerically higher chronic PTH and total alkaline phosphatase concentrations observed with lower dialysate calcium are biologically consistent with reduced calcium-mediated suppression of parathyroid secretion and increased skeletal turnover. Nonrandomized evidence evaluating lower dialysate calcium has reported similar changes in bone turnover markers, supporting the plausibility of this response [51]. However, an increase in PTH or alkaline phosphatase cannot be classified as beneficial or harmful without considering the baseline bone phenotype.

For patients with adynamic or low-turnover bone disease, moderate stimulation of PTH and bone formation may be desirable. In contrast, the same response could be unfavorable in patients with uncontrolled secondary hyperparathyroidism or pre-existing high-turnover disease. This phenotype dependence probably contributed to heterogeneity in the pooled PTH and alkaline phosphatase analyses, because the trials enrolled populations ranging from relative hypoparathyroidism and adynamic bone disease to secondary hyperparathyroidism.

Biochemical markers only partially characterize renal osteodystrophy. Comparisons with bone histology have shown that PTH, total alkaline phosphatase, bone-specific alkaline phosphatase, and collagen-turnover markers can help distinguish low from non-low turnover, but no individual marker fully reproduces the information obtained from bone biopsy [52,53]. Bone-specific alkaline phosphatase isoforms may provide additional information. The B1x isoform has been associated with low bone turnover, whereas higher bone-specific alkaline phosphatase and PTH are more consistent with non-low turnover [54]. Comparisons between bone-specific alkaline phosphatase and β-CrossLaps also support the complementary roles of formation and resorption markers rather than their use as interchangeable tests [55].

Phosphate kinetics further complicate interpretation. Phosphate removal during dialysis has been associated with markers of bone turnover, suggesting that skeletal flux and dialysis clearance are interrelated [56]. Serum C-terminal telopeptide has also been investigated as a marker of cortical bone loss in hemodialysis [57]. Consequently, treatment-related changes in PTH, alkaline phosphatase, phosphate, and resorption markers may reflect several simultaneous processes, including altered parathyroid secretion, skeletal uptake or release, dialysis clearance, and changes in concomitant therapy.

The prognostic importance of alkaline phosphatase reinforces the relevance of these biochemical changes but does not prove that treatment-induced alterations mediate clinical outcomes. Bone-specific and total alkaline phosphatase have been associated with mortality in dialysis cohorts [58,59,60]. More recent analyses suggest that longitudinal trajectories of PTH and alkaline phosphatase may be more informative than isolated measurements [61]. Future randomized trials should therefore evaluate repeated biochemical trends rather than relying exclusively on baseline and terminal values.

Bone-biopsy studies continue to demonstrate substantial heterogeneity in turnover, mineralization, and bone volume among patients with advanced CKD [62]. The histomorphometric improvement reported in one randomized dialysate-calcium trial is therefore clinically important but cannot be generalized to all dialysis populations. Trials directed at presumed low-turnover disease should use validated biochemical entry criteria and, where feasible, incorporate paired bone biopsies in a mechanistic subgroup.

### 4.4. Calcium Balance, Dialysis Modality, and Individualization

The acute analyses demonstrate that the dialysate-to-blood calcium gradient has an immediate and reproducible effect on postdialysis ionized calcium. Nevertheless, the calcium concentration printed on the dialysate prescription does not independently determine total calcium exposure. Net balance is also affected by predialysis ionized calcium, treatment duration, ultrafiltration, acid–base status, membrane characteristics, dialysis modality, and the concentrations of bicarbonate, magnesium, and other dialysate constituents.

This complexity supports individualized prescribing. Adjustment of dialysate calcium according to baseline predialysis calcium has been evaluated as an alternative to applying one fixed concentration to all patients [63]. Other approaches have combined ionized-calcium and PTH responses to identify sessions associated with excessive calcium gain or loss [64]. Acute kinetic studies similarly demonstrate that calcium transfer is dynamic and cannot be characterized fully by isolated predialysis and postdialysis total-calcium measurements [65].

Recent calcium-balance studies reinforce the importance of interpatient variability. Comparisons between hemodiafiltration and high-flux hemodialysis suggest that dialysate calcium concentration and treatment duration may be more influential determinants of sessional balance than modality alone, although modality-specific effects cannot be excluded [66]. Even with a dialysate calcium concentration of 1.25 mmol/L, some patients remain in positive balance, whereas others experience net calcium removal.

Long-term experience using more than one dialysate calcium concentration indicates that selective prescribing according to clinical and biochemical characteristics is feasible [67]. Dialysis frequency and session duration must also be considered. The cumulative weekly calcium exchange during quotidian, nocturnal, or other intensified schedules differs from that of conventional thrice-weekly dialysis, and a concentration appropriate for standard treatment may not be suitable for prolonged or frequent therapy [68].

These findings support a phenotype-based rather than concentration-based strategy. Patients with hypercalcemia, suppressed PTH, low-turnover bone disease, high calcium-binder exposure, or progressive vascular calcification may be candidates for a lower calcium prescription. Conversely, patients with recurrent intradialytic hypotension, symptomatic hypocalcemia, prolonged QTc, rapidly increasing PTH, high-turnover disease, or intensive dialysis schedules may require an intermediate or higher concentration. Such decisions should be based on serial trends and the complete treatment regimen rather than on one laboratory result.

### 4.5. The Bone–Vascular Trade-Off

Dialysate calcium cannot be evaluated solely as a skeletal intervention. Lower calcium may reduce calcium transfer and theoretically limit vascular mineral deposition, but it may also stimulate PTH, increase bone turnover, reduce vascular tone, and prolong ventricular repolarization. Higher calcium may improve intradialytic hemodynamic stability while promoting positive calcium balance and suppressing skeletal turnover. The relevant clinical question is therefore not whether lower or higher calcium is universally superior, but which prescription offers the most favorable balance for an individual patient.

The randomized coronary artery calcification evidence was inconsistent. Two compatible continuous estimates favored lower calcium, but the pooled interval was wide, while another randomized trial reported greater calcification progression with lower calcium. Differences in baseline calcification, bone turnover phenotype, PTH concentrations, phosphate-binder use, vitamin D exposure, follow-up duration, and outcome definition may explain part of this inconsistency. The current evidence cannot establish that lowering dialysate calcium consistently prevents or accelerates vascular calcification.

The pooled cIMT point estimate also favored lower calcium but remained imprecise. Arterial-stiffness outcomes could not be combined because the studies used different vascular segments, measurement methods, time points, and statistical models. Acute studies more consistently suggested that higher dialysate calcium increases blood pressure and arterial stiffness, but whether these transient changes translate into long-term cardiovascular benefit or harm remains uncertain.

An integrated bone–vascular framework is therefore needed. The ORCHESTRA cohort was designed to evaluate mineral metabolism, vascular calcification, and fracture within a shared dialysis population [69]. Population-based studies examining CKD–mineral and bone disorder monitoring and control similarly emphasize that biochemical management and clinical outcomes should be assessed longitudinally rather than as isolated cross-sectional targets [70].

This approach is particularly relevant because vascular calcification, osteoporosis, sarcopenia, inflammation, and malnutrition frequently coexist. A prescription chosen to improve one component may adversely affect another. Dialysate calcium should therefore be considered as one element of a multidimensional CKD–mineral and bone disorder strategy rather than an isolated exposure.

### 4.6. Clinical Implications

The findings support several principles for clinical practice. Lower dialysate calcium reliably lowers postdialysis ionized calcium and may increase acute or chronic PTH activity. It may also reduce postdialysis blood pressure and prolong QTc in susceptible patients. These physiological effects should be anticipated when the dialysate calcium gradient is reduced, particularly in patients with hemodynamic instability, arrhythmic risk, or limited capacity to tolerate acute calcium shifts.

At the same time, the randomized evidence is insufficient to prescribe lower dialysate calcium specifically to prevent fracture, preserve BMD, or reduce vascular calcification. Most outcome-specific analyses included only two to five small studies and generated wide modified Hartung–Knapp confidence intervals. Failure to detect a statistically significant pooled effect should therefore not be interpreted as proof of equivalence or absence of clinically important effects.

Prescription decisions should integrate serial total and, when relevant, ionized calcium; PTH, phosphate, and alkaline phosphatase trajectories; intradialytic symptoms and blood pressure responses; electrocardiographic risk; fracture history; BMD; vascular calcification; dialysis schedule; and concomitant medications. Dialysate calcium should be reassessed after major changes in vitamin D analogues, calcimimetics, phosphate binders, dialysis frequency, nutritional status, or residual kidney function. Accordingly, an identical dialysate calcium concentration may not represent an equivalent net calcium exposure across patients receiving different CKD–MBD therapies.

An intermediate calcium concentration may represent a reasonable initial prescription for many patients, followed by individualized adjustment. However, this review does not identify a universally preferred starting concentration or establish a new treatment threshold.

### 4.7. Strengths and Limitations

This review restricted its principal evidence base to randomized comparisons and linked multiple publications from the same population to prevent duplicate counting. Acute and chronic exposures were analyzed separately, as were total and ionized calcium, total and bone-specific alkaline phosphatase, arithmetic and geometric PTH measures, and clinically incompatible vascular outcomes. Crossover trials were analyzed using paired methods, factorial comparisons were selected within fixed levels of accompanying electrolytes whenever possible, and long-duration crossover BMD evidence was excluded from the primary synthesis because of potential carryover.

The statistical approach was adapted to sparse outcome-specific evidence. Random-effects models were combined with modified Hartung–Knapp inference, prediction intervals were calculated when estimable, and correlation assumptions were examined in sensitivity analyses. The reduction in acute ionized calcium remained robust across modality and leave-one-out analyses.

The review also has important limitations. Most syntheses contained few studies, and several estimates had wide confidence and prediction intervals. Many trials were small, single-center investigations, and several predated the widespread use of calcimimetics, non-calcium phosphate binders, and contemporary vitamin D receptor activator strategies. This temporal limitation is clinically important because these cointerventions can alter calcium loading, PTH suppression, and bone turnover and may therefore modify the biological response to a given dialysate calcium concentration. Pharmacologic exposure was incompletely and inconsistently reported across trials, and the available studies were too heterogeneous to determine whether calcimimetic use, phosphate-binder type, or vitamin D therapy modified the effect of dialysate calcium on net calcium balance or downstream skeletal and vascular outcomes. Consequently, the pooled estimates may not fully reflect calcium balance or treatment responses under contemporary CKD–MBD management. Populations also varied substantially in baseline PTH, bone turnover phenotype, vascular calcification, dialysis modality, treatment duration, and concomitant therapies.

Outcome reporting was heterogeneous. BMD was assessed using different technologies and anatomical sites, vascular outcomes were reported on incompatible scales, and adverse events were frequently recorded by dialysis session rather than by participant. Paired variances were often unavailable in crossover trials and required assumed correlations. Sensitivity analyses generally preserved the direction of the findings, but the precision of some estimates remained assumption-dependent.

In addition, dedicated trial registries and other sources specifically targeting unpublished or ongoing trials were not systematically searched. Although CENTRAL, citation tracking, companion publication screening, and publication status checks were used to identify additional reports, unpublished or ongoing randomized trials may therefore have been missed.

The review could not pool fractures, cardiovascular events, hospitalizations, or mortality as randomized outcomes because most trials focused on physiological or surrogate endpoints. Contextual observational studies demonstrate the prognostic importance of BMD, fracture, muscle status, PTH, and alkaline phosphatase, but they cannot establish that changing dialysate calcium improves these clinical outcomes.

Formal funnel-plot asymmetry testing was not performed because no homogeneous synthesis included at least 10 independent studies. Publication and selective-reporting bias therefore cannot be excluded. These limitations were incorporated into the outcome-specific risk-of-bias and GRADE certainty assessments, with most outcomes providing low- or very low-certainty evidence.

### 4.8. Future Research

Future randomized trials should move beyond fixed-concentration comparisons in unselected dialysis populations. Enrollment should be stratified by baseline total and ionized calcium, PTH, bone turnover phenotype, vascular calcification, fracture risk, cardiovascular stability, and dialysis modality. Each study should define whether it is primarily testing treatment of low-turnover disease, prevention of calcium loading, improvement of hemodynamic tolerance, or another specific therapeutic objective.

Long-term trials should include patient-important skeletal outcomes, including incident clinical and vertebral fractures, falls, site-specific DXA, volumetric BMD, trabecular measures, muscle strength, and physical performance. Mechanistic substudies should incorporate bone-specific alkaline phosphatase, validated resorption markers, and paired bone biopsies in selected participants. Vascular studies should use standardized coronary calcification and arterial stiffness measurements with blinded central interpretation and consistent reporting of absolute and relative progression.

Acute safety studies should include serial ionized-calcium measurements, continuous or repeated electrocardiographic assessment, intradialytic blood pressure, symptoms, and patient-level adverse-event reporting. Calcium-balance studies should document the complete dialysate composition, ionized dialysate calcium, ultrafiltration, session duration, modality, dietary calcium, phosphate binders, vitamin D therapy, and calcimimetic use.

Phenotype-guided or adaptive trials may be particularly informative. Rather than maintaining one concentration throughout follow-up, such studies could compare a standardized prescription with an algorithm that adjusts dialysate calcium according to serial ionized calcium, PTH, alkaline phosphatase, hemodynamic tolerance, and skeletal or vascular markers. This design would better reflect the individualized approach suggested by the current evidence and could determine whether improved biochemical and physiological targeting translates into fewer fractures, cardiovascular events, arrhythmias, hospitalizations, or deaths.

## 5. Conclusions

Lower dialysate calcium consistently decreases postdialysis ionized calcium and may increase parathyroid activity while reducing hemodynamic stability in susceptible patients. However, current randomized evidence does not establish a clear benefit for bone mineral density, fracture prevention, or vascular calcification, and most outcome-specific estimates remain imprecise. The balance between limiting calcium loading and avoiding hypocalcemia, secondary hyperparathyroidism, hypotension, and QTc prolongation is therefore likely to differ across patients. No single dialysate calcium concentration can be considered universally optimal. Prescription should be individualized according to serial mineral metabolism markers, bone turnover status, cardiovascular tolerance, dialysis modality, and concomitant therapy. Larger phenotype-guided randomized trials evaluating standardized skeletal, vascular, safety, and patient-important outcomes are needed.

## Figures and Tables

**Figure 1:**
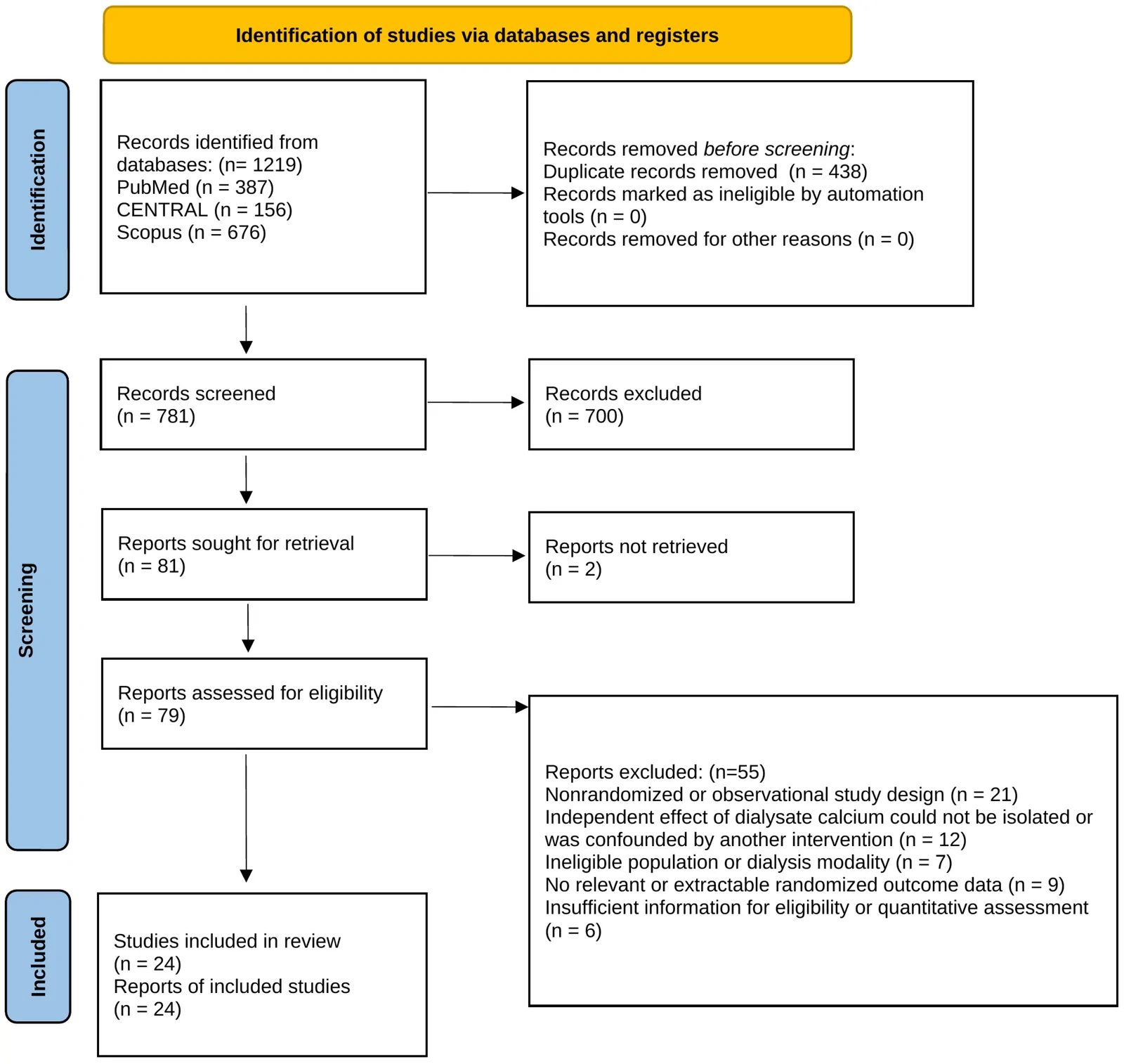
PRISMA 2020 flow diagram of study identification, screening, eligibility assessment, and inclusion in the systematic review and meta-analysis.

**Figure 3:**
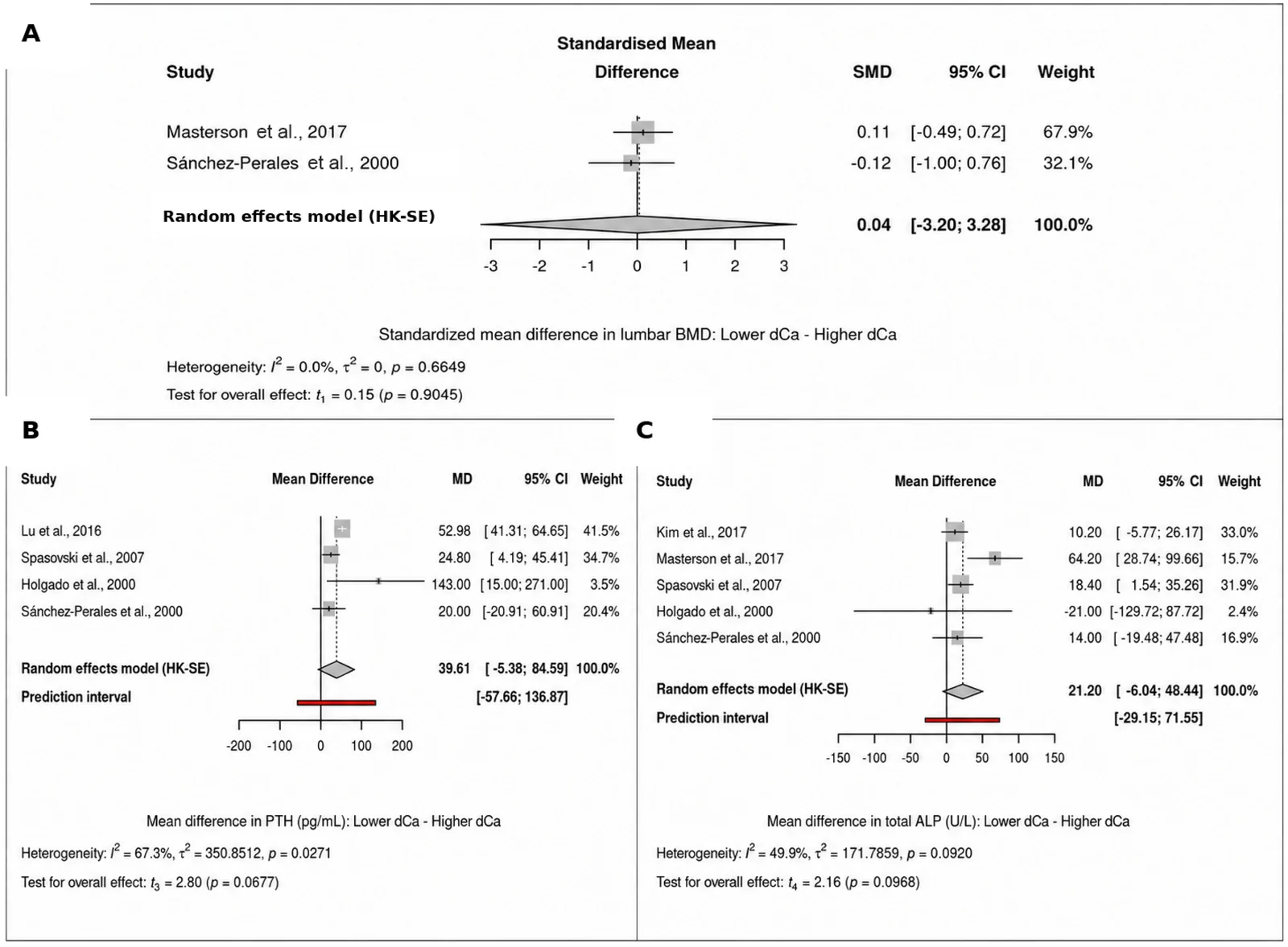
Bone-related outcomes comparing lower versus higher dialysate calcium concentrations. (**A**) Lumbar bone mineral density (BMD), expressed as standardized mean difference, including Masterson et al. (2017) [20] and Sánchez Perales et al. (2000) [38]. (**B**) Chronic parathyroid hormone (PTH), expressed as mean difference in pg/mL, including Lu et al. (2016) [18], Spasovski et al. (2007) [22], Holgado et al. (2000) [23], and Sánchez Perales et al. (2000) [38]. (**C**) Total alkaline phosphatase (ALP), expressed as mean difference in U/L, including Kim et al. (2017) [16], Masterson et al. (2017) [20], Spasovski et al. (2007) [22], Holgado et al. (2000) [23], and Sánchez Perales et al. (2000) [38]. Forest plots were generated using inverse-variance random-effects models with modified Hartung–Knapp confidence intervals; prediction intervals are shown where applicable.

**Figure 4:**
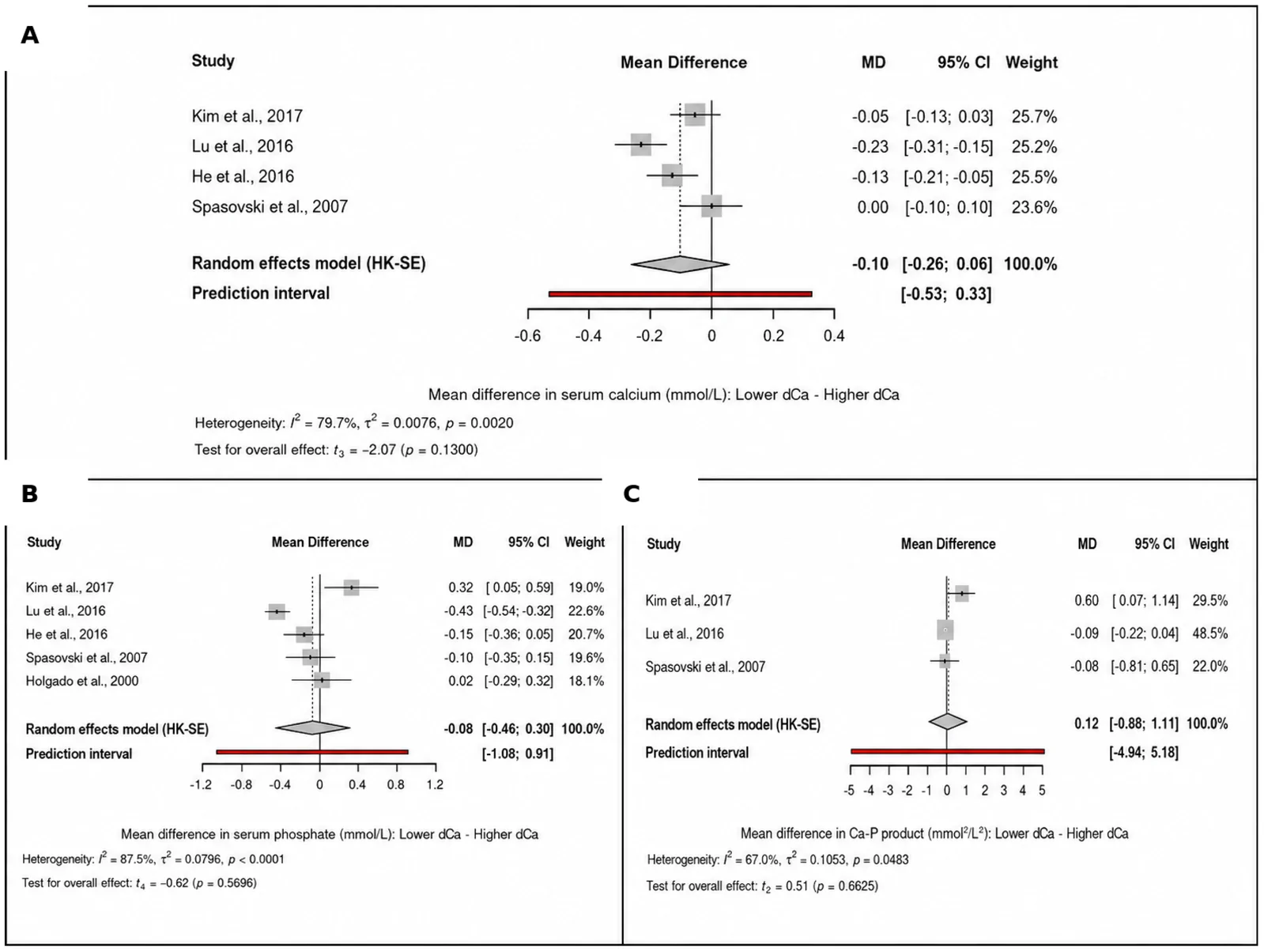
Chronic mineral metabolism outcomes comparing lower versus higher dialysate calcium concentrations. (**A**) Serum calcium, expressed as mean difference in mmol/L, including Kim et al. (2017) [16], Lu et al. (2016) [18], He et al. (2016) [19], and Spasovski et al. (2007) [22]. (**B**) Serum phosphate, expressed as mean difference in mmol/L, including Kim et al. (2017) [16], Lu et al. (2016) [18], He et al. (2016) [19], Spasovski et al. (2007) [22], and Holgado et al. (2000) [23]. (**C**) Calcium–phosphate product, expressed as mean difference in mmol^2^/L^2^, including Kim et al. (2017) [16], Lu et al. (2016) [18], and Spasovski et al. (2007) [22]. Forest plots were generated using inverse-variance random-effects models with modified Hartung–Knapp confidence intervals; prediction intervals are shown where applicable.

**Figure 5:**
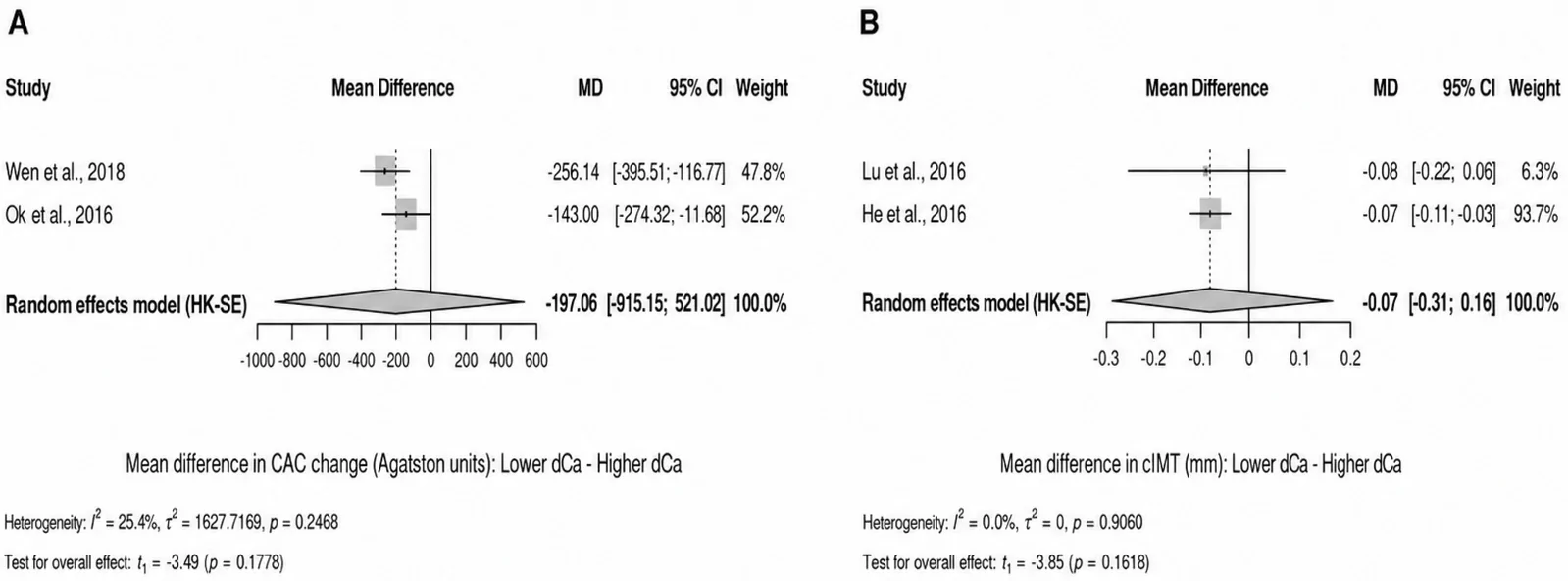
Vascular outcomes comparing lower versus higher dialysate calcium concentrations. (**A**) Change in coronary artery calcification (CAC), expressed as mean difference in Agatston units, including Wen et al. (2018) [15] and Ok et al. (2016) [17]. (**B**) Carotid intima–media thickness (cIMT), expressed as mean difference in mm, including Lu et al. (2016) [18] and He et al. (2016) [19]. Forest plots were generated using inverse-variance random-effects models with modified Hartung–Knapp confidence intervals.

**Figure 6:**
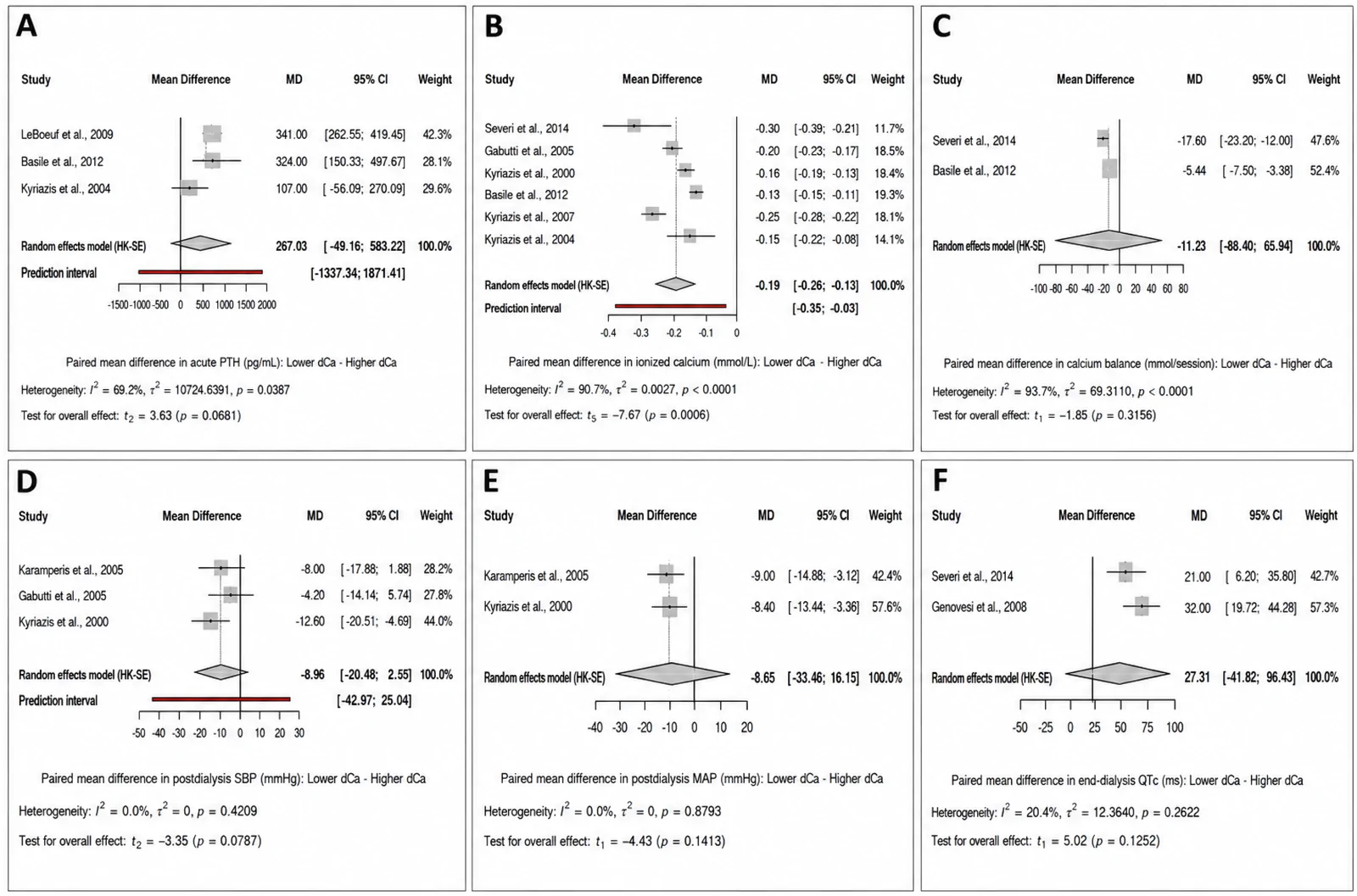
Acute physiological and safety outcomes comparing lower versus higher dialysate calcium concentrations. (**A**) Acute parathyroid hormone (PTH) response, expressed as paired mean difference in pg/mL, including LeBeouf et al. (2009) [24], Basile et al. (2012) [30], and Kyriazis et al. (2004) [34]. (**B**) End-of-dialysis ionized calcium, expressed as paired mean difference in mmol/L, including Severi et al. (2014) [25], Gabutti et al. (2005) [28], Kyriazis et al. (2000) [29], Basile et al. (2012) [30], Kyriazis et al. (2007) [31], and Kyriazis et al. (2004) [34]. (**C**) Calcium mass balance, expressed as paired mean difference in mmol/session, including Severi et al. (2014) [25] and Basile et al. (2012) [30]. (**D**) Postdialysis systolic blood pressure (SBP), expressed as paired mean difference in mmHg, including Karamperis et al. (2005) [27], Gabutti et al. (2005) [28], and Kyriazis et al. (2000) [29]. (**E**) Postdialysis mean arterial pressure (MAP), expressed as paired mean difference in mmHg, including Karamperis et al. (2005) [27] and Kyriazis et al. (2000) [29]. (**F**) End-of-dialysis corrected QT (QTc) interval, expressed as paired mean difference in ms, including Severi et al. (2014) [25] and Genovesi et al. (2008) [35]. Effect estimates are calculated as the value with lower dialysate calcium minus the value with higher dialysate calcium. Forest plots use inverse-variance random-effects models with DerSimonian–Laird estimation of between-study variance and modified Hartung–Knapp confidence intervals. Prediction intervals are shown for syntheses containing at least three clinically comparable studies where estimable.

**Table 1:** Characteristics of randomized trials included in the systematic review and meta-analysis.

Study	Design	Dialysis Modality	Dialysate/Infusate Calcium Strategy	Randomized/Enrolled N	Primary Analyzed N	Duration	Principal Outcomes/Role in Review
**Wen et al., 2018** [15]	Parallel RCT	Conventional HD	1.25 vs. 1.50 mmol/L	86 vs. 88	80 vs. 84 for CAC/completion	12 months	CAC progression; chronic biochemical/vascular outcomes; safety
**Kim et al., 2017** [16]	Open-label parallel RCT	Conventional HD	1.25 vs. 1.50 mmol/L	36 vs. 40	30 vs. 34 at 12 months	12 months	CAC; serum calcium; phosphate; Ca × P; ALP
**Ok et al., 2016** [17]	Parallel RCT	Conventional HD	1.25 vs. 1.75 mmol/L	212 vs. 213	CAC paired subset: 120 vs. 104; paired biopsy: 55 vs. 53	24 months	CAC; bone histomorphometry; chronic mineral metabolism
**Lu et al., 2016** [18]	Parallel RCT	Conventional HD	1.25 vs. 1.50 mmol/L	41 vs. 41	35 vs. 38 at month 12	12 months	PTH; calcium; phosphate; Ca × P; cIMT
**He et al., 2016** [19]	Parallel RCT	Conventional HD	1.25 vs. 1.50 mmol/L	64 vs. 64	59 vs. 51 at month 24	24 months	Calcium/phosphate metabolism; cIMT; aortic stiffness
**Masterson et al., 2017** [20]	Parallel RCT	Alternate-night nocturnal HD	1.30 vs. 1.60 or 1.75 mmol/L according to center	24 vs. 26	22 vs. 20 for outcome analysis	12 months	BMD; ALP; chronic mineral metabolism outcomes
**LeBoeuf et al., 2011** [21]	Parallel RCT	Conventional HD	1.12 vs. 1.37 mmol/L	30 total	14 vs. 13 in intervention-period analysis	6 months	Aortic stiffness; chronic vascular/mechanistic outcomes
**Spasovski et al., 2007** [22]	Parallel RCT	Conventional HD	1.25 vs. 1.75 mmol/L	30 vs. 30	26 vs. 26 completed	6 months	Bone turnover; PTH; ALP; calcium; phosphate; Ca × P
**Holgado et al., 2000** [23]	Parallel RCT	Conventional HD	Low 2.25/2.50 vs. regular 3.00/3.50 mEq/L, center-dependent	17 vs. 16	11 vs. 10 at 12 months	12 months	PTH; ALP; phosphate; chronic mineral metabolism
**LeBeouf et al., 2009** [24]	Randomized Latin-square crossover	Conventional HD	1.00, 1.25, and 1.50 mmol/L	18 crossover	18	Single sessions	Acute PTH response; arterial stiffness; aortic pressure
**Severi et al., 2014** [25]	Randomized crossover	HDF	1.25, 2.00 mmol/L and profiled calcium	22 crossover	22	Three sessions	Ionized calcium; calcium balance; QTc; acute safety
**Gabutti et al., 2009** [26]	Randomized single-blind factorial crossover	Conventional HD	1.25 vs. 1.50 mmol/L with bicarbonate variation	21 crossover	21	Repeated sessions	Acute hemodynamic and electrolyte responses
**Karamperis et al., 2005** [27]	Randomized blinded crossover	Predilution hemofiltration	Infusate 1.25 vs. 1.75 mmol/L	12 crossover	12	Two sessions	Ionized calcium; SBP; MAP; acute hemodynamics
**Gabutti et al., 2005** [28]	Randomized single-blind crossover	Conventional HD	1.25 vs. 1.50 mmol/L at fixed bicarbonate 32 mmol/L	27 crossover	27	Three 3-week modalities	Ionized calcium; blood pressure; acute hemodynamics
**Kyriazis et al., 2000** [29]	Randomized crossover	Conventional HD	1.25 vs. 1.75 mmol/L	19 crossover	19	Four sessions per period	Ionized calcium; SBP; MAP; arterial compliance
**Basile et al., 2012** [30]	Randomized three-period crossover	Conventional HD	1.25, 1.375, and 1.50 mmol/L	22 crossover	22; PTH n = 19	Three sessions	Ionized calcium; acute PTH; calcium mass balance
**Kyriazis et al., 2007** [31]	Randomized crossover	Conventional HD	1.25 vs. 1.75 mmol/L	14 crossover	14	Two sessions	Ionized calcium; arterial stiffness
**Kyriazis et al., 2002** [32]	Partly randomized crossover	Conventional HD	Low constant vs. calcium-profile treatment randomized; medium constant treatment subsequently administered	Study A: 18; Study B: 8	18 and 8	Single sessions/3-week periods	Dialysate-calcium profiling and acute/chronic physiological responses
**Sonikian et al., 2009** [33]	Randomized three-way crossover	Conventional HD	1.25, 1.50, and 1.75 mmol/L	12 crossover	12	2–14 weeks per period	PTH and mineral metabolism during paricalcitol/sevelamer treatment
**Kyriazis et al., 2004** [34]	Randomized 2 × 2 factorial crossover	Conventional HD	Ca 1.25/1.75 × Mg 0.25/0.75 mmol/L	8 crossover	8	Four sessions	Ionized calcium; acute PTH; blood pressure physiology
**Genovesi et al., 2008** [35]	Randomized 2 × 3 factorial crossover	Conventional HD	Ca 1.25/1.50/1.75 × K 2/3 mmol/L	16 crossover	16	Six sessions	QT/QTc; electrophysiological safety
**Di Iorio et al., 2012** [36]	Randomized blinded factorial crossover	Conventional HD	Ca 1.25/1.50/1.75 mmol/L with potassium and bicarbonate variation	22 crossover	22	Multiple sessions	QTc; acute mineral/electrophysiological outcomes
**Van der Niepen et al., 1995** [37]	Randomized two-period crossover	Conventional HD	1.25 vs. 1.75 mmol/L	19 selected; 14 completed	14 crossover	6 months per period	BMD and chronic skeletal outcomes
**Sánchez Perales et al., 2000** [38]	Parallel RCT	Conventional HD	1.25 vs. 1.50 mmol/L	11 vs. 9	11 vs. 9	12 months	QCT BMD; PTH; ALP; chronic mineral outcomes

Notes: For crossover and factorial trials, the reported sample size represents participants exposed to multiple randomized treatment conditions rather than independent parallel groups. Primary analyzed sample sizes are outcome-specific where attrition or dedicated imaging/biopsy subsets occurred. Calcium concentrations are reported in mmol/L unless the original study reported mEq/L. Methodological note: For Kyriazis et al. [32], only the randomized comparison was considered randomized evidence for synthesis; nonrandomized treatment contrasts were not included in the strict randomized analyses. Abbreviations: ALP, alkaline phosphatase; BMD, bone mineral density; CAC, coronary artery calcification; Ca × P, calcium–phosphate product; cIMT, carotid intima–media thickness; HD, hemodialysis; HDF, hemodiafiltration; MAP, mean arterial pressure; PTH, parathyroid hormone; QCT, quantitative computed tomography; QTc, corrected QT interval; RCT, randomized controlled trial; SBP, systolic blood pressure.

**Table 2:** GRADE assessment of the certainty of the evidence for principal outcomes comparing lower versus higher dialysate calcium concentrations.

Outcome	Studies/Participants	Effect Estimate (95% CI)	Pooled Absolute Effect	Certainty of Evidence	Interpretation
Lumbar BMD	2 randomized trials/62 participants	SMD 0.04 (−3.20 to 3.28)	No clear difference in lumbar BMD	Very low (a,b,c)	Lower dCa did not demonstrate a clear effect on lumbar BMD. The confidence interval was extremely wide and compatible with substantial benefit, no effect, or harm.
Chronic PTH	4 randomized trials/166 participants	MD 39.61 pg/mL (−5.38 to 84.59)	Lower dCa associated with 39.61 pg/mL higher PTH, but CI crossed no effect	Very low (a,d,e)	Lower dCa may increase chronic PTH, but the evidence is very uncertain because heterogeneity and imprecision were substantial.
Total ALP	5 randomized trials/199 participants	MD 21.20 U/L (−6.04 to 48.44)	Lower dCa associated with 21.20 U/L higher ALP, but CI crossed no effect	Very low (a,d,e)	No clear difference in total ALP was demonstrated. The available evidence remains very uncertain.
Serum calcium	4 randomized trials/299 participants	MD −0.10 mmol/L (−0.26 to 0.06)	Lower dCa associated with slightly lower serum calcium, but CI crossed no effect	Very low (a,d,e)	Lower dCa may reduce serum calcium slightly, but the pooled estimate is imprecise and inconsistent across studies.
Serum phosphate	5 randomized trials/320 participants	MD −0.08 mmol/L (−0.46 to 0.30)	Lower dCa associated with slightly lower serum phosphate, but CI crossed no effect	Very low (a,d,e)	No clear difference in serum phosphate was detected.
Calcium–phosphate product	3 randomized trials/189 participants	MD 0.12 mmol^2^/L^2^ (−0.88 to 1.11)	No clear difference in Ca–P product	Very low (a,d,c)	Evidence was too imprecise to determine whether lower dCa affects Ca–P product.
Coronary artery calcification (CAC) progression	2 randomized trials/388 participants	MD −197.06 Agatston units (−915.15 to 521.02)	Lower dCa associated with less CAC progression on average, but CI was very wide	Very low (f,c)	Lower dCa may reduce CAC progression, but the evidence is highly uncertain and does not permit a reliable conclusion.
Carotid intima–media thickness (cIMT)	2 randomized trials/183 participants	MD −0.07 mm (−0.31 to 0.16)	No clear difference in cIMT	Low (c)	No clear effect of lower dCa on cIMT was demonstrated, although the direction of effect favored lower dCa.
End-dialysis ionized calcium	6 crossover randomized trials/112 participants	MD −0.19 mmol/L (−0.26 to −0.13)	Lower dCa reduced end-dialysis ionized calcium by 0.19 mmol/L	Moderate (d)	Lower dCa probably reduces end-dialysis ionized calcium. This was the most consistent and robust finding in the review.
Calcium balance	2 crossover randomized trials/44 participants	MD −11.23 mmol/session (−88.40 to 65.94)	No clear difference in calcium balance	Very low (a,d,c)	Evidence was too uncertain to determine whether lower dCa changes calcium balance.
Acute PTH response	3 crossover randomized trials/45 participants	MD 267.03 pg/mL (−49.16 to 583.22)	Lower dCa associated with higher acute PTH response, but CI crossed no effect	Very low (a,d,c)	Lower dCa may stimulate acute PTH release, but the evidence is very uncertain.
Postdialysis systolic blood pressure	3 crossover randomized trials/58 participants	MD −8.97 mmHg (−20.48 to 2.55)	Lower dCa associated with lower postdialysis SBP, but CI crossed no effect	Low (a,e)	Lower dCa may reduce postdialysis SBP, but the estimate is imprecise and not definitive.
Postdialysis mean arterial pressure	2 crossover randomized trials/31 participants	MD −8.65 mmHg (−33.45 to 16.15)	No clear difference in postdialysis MAP	Very low (a,c)	Evidence was too limited and imprecise to determine whether lower dCa affects postdialysis MAP.
End-dialysis QTc	2 crossover randomized trials/38 participants	MD 27.31 ms (−41.82 to 96.43)	No clear difference in end-dialysis QTc	Very low (a,c)	Evidence was too uncertain to determine whether lower dCa prolongs QTc at the end of dialysis.

**a** Downgraded one level for risk of bias because of limitations in reporting of randomization, allocation concealment, blinding, prespecified analyses, or crossover-specific safeguards in contributing trials. **b** Downgraded one level for indirectness because the BMD evidence was derived from only two clinically and methodologically different studies, including alternate-night nocturnal hemodialysis. **c** Downgraded two levels for very serious imprecision because the confidence interval was very wide and compatible with materially different clinical conclusions. **d** Downgraded one level for inconsistency because of substantial between-study heterogeneity in effect magnitude. **e** Downgraded one level for serious imprecision because the confidence interval crossed the line of no effect and remained compatible with clinically meaningful effects. **f** Downgraded for inconsistency because continuous pooled CAC findings and other randomized CAC findings did not provide a consistent direction of effect. Risk-of-bias judgments within GRADE were based on the outcome-specific RoB 2 assessments presented in Supplementary Table S3. Because GRADE evaluates risk of bias across the body of evidence for an outcome, an individual RoB 2 judgment of “some concerns” did not automatically result in downgrading; downgrading was applied when the identified limitations were judged sufficiently important to reduce confidence in the pooled outcome estimate. **Abbreviations**: ALP, alkaline phosphatase; BMD, bone mineral density; CAC, coronary artery calcification; CI, confidence interval; cIMT, carotid intima–media thickness; dCa, dialysate calcium; MAP, mean arterial pressure; MD, mean difference; PTH, parathyroid hormone; QTc, corrected QT interval; SBP, systolic blood pressure; SMD, standardized mean difference.

## Data Availability

All data analyzed in this systematic review were derived from published studies cited in the reference list. The Supplementary Materials include the complete database search strategies, REML sensitivity analyses, outcome-specific risk-of-bias assessments, and the modality-inclusive ionized-calcium sensitivity analysis. No individual-participant data were used.

## Supplementary Materials

The following supporting information can be downloaded at https://www.mdpi.com/article/10.3390/diagnostics16193181/s1: Table S1: Complete electronic search strategies for PubMed/MEDLINE, CENTRAL, and Scopus; Table S2: Sensitivity of pooled estimates to restricted maximum likelihood estimation of between-study variance; Table S3: Outcome-specific Cochrane Risk of Bias 2 assessments for results contributing to the principal quantitative syntheses; Figure S1: Sensitivity analysis of end-dialysis ionized calcium including all eligible dialysis modalities.

## Abbreviations

The following abbreviations are used in this manuscript:

ALP	Alkaline phosphatase
BMD	Bone mineral density
CAC	Coronary artery calcification
Ca × P	Calcium–phosphate product
CENTRAL	Cochrane Central Register of Controlled Trials
CI	Confidence interval
cIMT	Carotid intima–media thickness
CKD	Chronic kidney disease
CKD–MBD	Chronic kidney disease–mineral and bone disorder
CTX	C-terminal telopeptide of type I collagen
dCa	Dialysate calcium
DXA	Dual-energy X-ray absorptiometry
GRADE	Grading of Recommendations Assessment, Development and Evaluation
HD	Hemodialysis
HDF	Hemodiafiltration
MAP	Mean arterial pressure
MD	Mean difference
P1NP	Procollagen type I N-terminal propeptide
PRISMA	Preferred Reporting Items for Systematic Reviews and Meta-Analyses
PROSPERO	International Prospective Register of Systematic Reviews
PTH	Parathyroid hormone
QCT	Quantitative computed tomography
QT	QT interval
QTc	Corrected QT interval
RCT	Randomized controlled trial
RoB 2	Cochrane Risk of Bias 2
SBP	Systolic blood pressure
SMD	Standardized mean difference
