# Supplementary material for: Effects of Dialysate Calcium Concentration on Bone, Mineral Metabolism, and Vascular Outcomes in Maintenance Hemodialysis: A Systematic Review and Meta-Analysis
Source: Diagnostics (Basel). 2026 Sep 30;16(19):3181. doi: 10.3390/diagnostics16193181 (PMC13664609; doi:10.3390/diagnostics16193181)
Supplement: Supplementary file 1 [file diagnostics-16-03181-s001.zip › Supplementary_Table_S1.pdf]

Supplementary Table S1. Complete electronic search strategies for PubMed/MEDLINE, CENTRAL, and Scopus.

Pubmed:

```
(
 "Renal Dialysis"[Mesh]
 OR hemodialysis[tiab]
 OR haemodialysis[tiab]
 OR hemodiafiltration[tiab]
 OR haemodiafiltration[tiab]
 OR hemofiltration[tiab]
 OR haemofiltration[tiab]
)
AND
(
 "dialysate calcium"[tiab]
 OR "calcium dialysate"[tiab]
 OR "calcium profiling"[tiab]
 OR
 (
  calcium[tiab]
  AND
  (
   dialysate[tiab]
   OR "dialysis fluid"[tiab]
   OR "dialysis solution"[tiab]
   OR "replacement fluid"[tiab]
   OR infusate[tiab]
  )
 )
```

)

AND

(

random*[tiab]

OR trial[tiab]

OR crossover[tiab]

OR "cross-over"[tiab]

OR prospective[tiab]

OR intervention*[tiab]

OR comparative[tiab]

OR controlled[tiab]

OR clinical study[pt]

OR clinical trial[pt]

)

Central:

#1 (hemodialysis OR haemodialysis OR hemodiafiltration OR haemodiafiltration OR hemofiltration OR haemofiltration):ti,ab,kw

#2 ("dialysate calcium" OR "calcium dialysate" OR "calcium profiling"):ti,ab,kw

#3 ((dialysate OR "dialysis fluid" OR "replacement fluid" OR infusate) near/3 calcium):ti,ab,kw

#4 #2 OR #3

#5 #1 AND #4

Scopus:

TITLE-ABS-KEY(

(hemodialysis OR haemodialysis

OR hemodiafiltration OR haemodiafiltration

OR hemofiltration OR haemofiltration)

AND

(

"dialysate calcium"

OR "calcium dialysate"

OR "calcium profiling"

OR (dialysate W/3 calcium)

OR ("dialysis fluid" W/3 calcium)

OR ("dialysis solution" W/3 calcium)

OR ("replacement fluid" W/3 calcium)

OR (infusate W/3 calcium)

)

)
