# Supplementary material for: Effects of Dialysate Calcium Concentration on Bone, Mineral Metabolism, and Vascular Outcomes in Maintenance Hemodialysis: A Systematic Review and Meta-Analysis
Source: Diagnostics (Basel). 2026 Sep 30;16(19):3181. doi: 10.3390/diagnostics16193181 (PMC13664609; doi:10.3390/diagnostics16193181)
Supplement: Supplementary file 1 [file diagnostics-16-03181-s001.zip › Supplementary_Table_S2_REML_Sensitivity.pdf]

Supplementary Materials

Effects of Dialysate Calcium Concentration on Bone, Mineral Metabolism, and Vascular Outcomes in Maintenance Hemodialysis: A Systematic Review and Meta-Analysis

Supplementary Methods - REML sensitivity analysis

All primary quantitative syntheses were repeated using restricted maximum likelihood (REML) estimation of between-study variance. Study inclusion, effect measures, effect direction (lower dialysate calcium minus higher dialysate calcium), variance reconstruction, crossover assumptions, and the modified Hartung-Knapp standard-error safeguard used in the primary analyses were otherwise retained. I-squared is shown once because it is based on Cochran's Q and does not change when only the tau-squared estimator is changed.

Supplementary Table S2. Sensitivity of pooled estimates to REML estimation of between-study variance.

Outcome	k	Effect measure	Primary DL + mHK estimate (95% CI)	I^2 (%)	DL τ^2	REML + mHK estimate (95% CI)	REML τ^2	Interpretation
Lumbar BMD	2	SMD (Hedges' g)	0.04 (-3.20 to 3.28)	0.0	0	0.04 (-3.20 to 3.28)	0	Unchanged; no clear difference
Chronic PTH (pg/mL)	4	MD	39.61 (-5.38 to 84.59)	67.3	350.8512	39.61 (-2.53 to 81.75)	253.4936	Unchanged; higher point estimate, CI crosses null
Total ALP (U/L)	5	MD	21.20 (-6.04 to 48.44)	49.9	171.7859	21.54 (-6.46 to 49.53)	219.0865	Unchanged; higher point estimate, CI crosses null
Serum calcium (mmol/L)	4	MD	-0.103 (-0.262 to 0.055)	79.7	0.0076	-0.105 (-0.264 to 0.054)	0.0080	Unchanged; lower point estimate, CI crosses null
Serum phosphate (mmol/L)	5	MD	-0.085 (-0.465 to 0.295)	87.5	0.0796	-0.086 (-0.437 to 0.264)	0.0657	Unchanged; no clear difference
Calcium-phosphate product ($mmol^2/L^2$)	3	MD	0.117 (-0.876 to 1.110)	67.0	0.1053	0.115 (-0.861 to 1.091)	0.1004	Unchanged; no clear difference
Coronary artery calcification change (Agatston units)	2	MD	-197.06 (-915.15 to 521.02)	25.4	1627.7169	-197.06 (-915.15 to 521.02)	1627.7200	Unchanged; imprecise, CI crosses null
Carotid intima-media thickness (mm)	2	MD	-0.072 (-0.308 to 0.165)	0.0	0	-0.072 (-0.308 to 0.165)	0	Unchanged; no clear difference
Acute PTH response (pg/mL)	3	Paired MD	267.03 (-49.16 to 583.22)	69.2	10724.6391	267.03 (-49.16 to 583.22)	10724.7904	Unchanged; greater PTH response, CI crosses null
End-dialysis ionized calcium (mmol/L)	6	Paired MD	-0.193 (-0.258 to -0.128)	90.7	0.0027	-0.193 (-0.258 to -0.128)	0.0029	Unchanged; clear reduction remains
Calcium mass balance (mmol/session)	2	Paired MD	-11.23 (-88.40 to 65.94)	93.7	69.3110	-11.23 (-88.40 to 65.94)	69.2989	Unchanged; CI crosses null
Postdialysis SBP (mmHg)	3	Paired MD	-8.97 (-20.48 to 2.55)	0.0	0	-8.97 (-20.48 to 2.55)	0	Unchanged; lower point estimate, CI crosses null
Postdialysis MAP (mmHg)	2	Paired MD	-8.65 (-33.46 to 16.15)	0.0	0	-8.65 (-33.46 to 16.15)	0	Unchanged; lower point estimate, CI crosses null
End-dialysis QTc (ms)	2	Paired MD	27.31 (-41.82 to 96.43)	20.4	12.3640	27.31 (-41.82 to 96.43)	12.3642	Unchanged; longer point estimate, CI crosses null

Notes: Effects are lower dCa minus higher dCa. DL = DerSimonian-Laird; REML = restricted maximum likelihood; mHK = modified Hartung-Knapp; MD = mean difference; SMD = standardized mean difference. For lumbar BMD, cIMT, SBP, and MAP, both estimators gave tau-squared = 0, so the pooled estimate and safeguarded CI were unchanged. The REML prediction interval for end-dialysis ionized calcium remained below the null (-0.345 to -0.041 mmol/L).

Overall: Across all 14 primary quantitative syntheses, replacing DL with REML did not materially change the direction or overall interpretation of the pooled findings. End-dialysis ionized calcium remained the only pooled outcome with both its confidence interval and prediction interval entirely below the null.
