# Supplementary material for: Effects of Dialysate Calcium Concentration on Bone, Mineral Metabolism, and Vascular Outcomes in Maintenance Hemodialysis: A Systematic Review and Meta-Analysis
Source: Diagnostics (Basel). 2026 Sep 30;16(19):3181. doi: 10.3390/diagnostics16193181 (PMC13664609; doi:10.3390/diagnostics16193181)
Supplement: Supplementary file 1 [file diagnostics-16-03181-s001.zip › Supplementary_Table_S3_Outcome_Specific_RoB2_FINAL.pdf]

Supplementary Table S3. Outcome-specific Cochrane RoB 2 assessments for results contributing to the principal quantitative syntheses

This table expands the study-level overview shown in Figure 2 by identifying the specific outcome/result contributed by each trial to the principal quantitative syntheses and GRADE assessments. Where a trial contributed more than one principal outcome and the documented result-level domain judgment did not differ, the same RoB 2 profile is repeated across those outcomes. For crossover trials, period and carryover considerations were incorporated into the harmonized domain judgments, consistent with the approach described in the manuscript.

Outcome/result	Study	D1	D2	D3	D4	D5	Overall	GRADE RoB impact
Lumbar BMD	Masterson et al., 2017 [20]	L	L	SC	L	L	SC	Serious (-1)
Lumbar BMD	Sanchez Perales et al., 2000 [38]	SC	SC	L	L	SC	SC	Serious (-1)
Chronic PTH	Lu et al., 2016 [18]	SC	L	SC	L	SC	SC	Serious (-1)
Chronic PTH	Spasovski et al., 2007 [22]	SC	L	SC	L	SC	SC	Serious (-1)
Chronic PTH	Holgado et al., 2000 [23]	SC	SC	SC	L	SC	SC	Serious (-1)
Chronic PTH	Sanchez Perales et al., 2000 [38]	SC	SC	L	L	SC	SC	Serious (-1)
Total ALP	Kim et al., 2017 [16]	SC	SC	SC	L	SC	SC	Serious (-1)
Total ALP	Masterson et al., 2017 [20]	L	L	SC	L	L	SC	Serious (-1)
Total ALP	Spasovski et al., 2007 [22]	SC	L	SC	L	SC	SC	Serious (-1)
Total ALP	Holgado et al., 2000 [23]	SC	SC	SC	L	SC	SC	Serious (-1)
Total ALP	Sanchez Perales et al., 2000 [38]	SC	SC	L	L	SC	SC	Serious (-1)
Serum calcium	Kim et al., 2017 [16]	SC	SC	SC	L	SC	SC	Serious (-1)
Serum calcium	Lu et al., 2016 [18]	SC	L	SC	L	SC	SC	Serious (-1)
Serum calcium	He et al., 2016 [19]	SC	SC	SC	L	SC	SC	Serious (-1)
Serum calcium	Spasovski et al., 2007 [22]	SC	L	SC	L	SC	SC	Serious (-1)
Serum phosphate	Kim et al., 2017 [16]	SC	SC	SC	L	SC	SC	Serious (-1)
Serum phosphate	Lu et al., 2016 [18]	SC	L	SC	L	SC	SC	Serious (-1)
Serum phosphate	He et al., 2016 [19]	SC	SC	SC	L	SC	SC	Serious (-1)
Serum phosphate	Spasovski et al., 2007 [22]	SC	L	SC	L	SC	SC	Serious (-1)
Serum phosphate	Holgado et al., 2000 [23]	SC	SC	SC	L	SC	SC	Serious (-1)
Calcium-phosphate product	Kim et al., 2017 [16]	SC	SC	SC	L	SC	SC	Serious (-1)
Calcium-phosphate product	Lu et al., 2016 [18]	SC	L	SC	L	SC	SC	Serious (-1)
Calcium-phosphate product	Spasovski et al., 2007 [22]	SC	L	SC	L	SC	SC	Serious (-1)
Coronary artery calcification	Wen et al., 2018 [15]	SC	L	L	L	SC	SC	Not serious
Coronary artery calcification	Ok et al., 2016 [17]	SC	SC	SC	L	SC	SC	Not serious
Carotid intima-media thickness	Lu et al., 2016 [18]	SC	L	SC	L	SC	SC	Not serious
Carotid intima-media thickness	He et al., 2016 [19]	SC	SC	SC	L	SC	SC	Not serious
End-dialysis ionized calcium	Severi et al., 2014 [25]	SC	SC	L	L	SC	SC	Not serious
End-dialysis ionized calcium	Gabutti et al., 2005 [28]	L	SC	L	L	SC	SC	Not serious
End-dialysis ionized calcium	Kyriazis et al., 2000 [29]	SC	SC	L	SC	SC	SC	Not serious
End-dialysis ionized calcium	Basile et al., 2012 [30]	SC	L	L	L	SC	SC	Not serious
End-dialysis ionized calcium	Kyriazis et al., 2007 [31]	SC	SC	L	SC	SC	SC	Not serious
End-dialysis ionized calcium	Kyriazis et al., 2004 [34]	SC	L	L	L	SC	SC	Not serious
Calcium mass balance	Severi et al., 2014 [25]	SC	SC	L	L	SC	SC	Serious (-1)
Calcium mass balance	Basile et al., 2012 [30]	SC	L	L	L	SC	SC	Serious (-1)
Acute PTH response	LeBoeuf et al., 2009 [24]	SC	L	SC	L	SC	SC	Serious (-1)
Acute PTH response	Basile et al., 2012 [30]	SC	L	L	L	SC	SC	Serious (-1)
Acute PTH response	Kyriazis et al., 2004 [34]	SC	L	L	L	SC	SC	Serious (-1)
Postdialysis systolic BP	Karamperis et al., 2005 [27]	SC	L	L	L	SC	SC	Serious (-1)
Postdialysis systolic BP	Gabutti et al., 2005 [28]	L	SC	L	L	SC	SC	Serious (-1)
Postdialysis systolic BP	Kyriazis et al., 2000 [29]	SC	SC	L	SC	SC	SC	Serious (-1)
Postdialysis mean arterial pressure	Karamperis et al., 2005 [27]	SC	L	L	L	SC	SC	Serious (-1)
Postdialysis mean arterial pressure	Kyriazis et al., 2000 [29]	SC	SC	L	SC	SC	SC	Serious (-1)
End-dialysis QTc	Severi et al., 2014 [25]	SC	SC	L	L	SC	SC	Serious (-1)
End-dialysis QTc	Genovesi et al., 2008 [35]	SC	SC	L	L	SC	SC	Serious (-1)

Abbreviations and domain definitions: L, low risk of bias; SC, some concerns; BMD, bone mineral density; PTH, parathyroid hormone; ALP, alkaline phosphatase; BP, blood pressure; QTc, corrected QT interval. D1, bias arising from the randomization process; D2, bias due to deviations from intended interventions; D3, bias due to missing outcome data; D4, bias in measurement of the outcome; D5, bias in selection of the reported result.

GRADE linkage: “Serious (-1)” indicates that the outcome-level GRADE assessment in Table 2 was downgraded one level for risk of bias. “Not serious” indicates no GRADE downgrade for risk of bias, despite some concerns in individual RoB 2 domains.

Scope: Table S3 covers the study results contributing to the principal quantitative syntheses and corresponding GRADE judgments. Figure 2 remains the concise study-level overview for all 24 included randomized trials.
